# Effect of SSR504734, a Selective Glycine Transporter Type 1 Inhibitor, on Seizure Thresholds, Neurotransmitter Levels, and Inflammatory Markers in Mice

**DOI:** 10.1021/acschemneuro.5c00039

**Published:** 2025-02-27

**Authors:** Nikola Gapińska, Piotr Wlaź, Elżbieta Wyska, Artur Świerczek, Krzysztof Kamiński, Marcin Jakubiec, Michał Abram, Katarzyna Ciepiela, Gniewomir Latacz, Tymoteusz Słowik, Dawid Krokowski, Łukasz Jarosz, Artur Ciszewski, Katarzyna Socała

**Affiliations:** † Department of Animal Physiology and Pharmacology, Institute of Biological Sciences, Maria Curie-Skłodowska University, Akademicka 19, 20-033 Lublin, Poland; ‡ Doctoral School of Quantitative and Natural Sciences, Maria Curie-Skłodowska University, Weteranów 18, 20-038 Lublin, Poland; § Department of Pharmacokinetics and Physical Pharmacy, Faculty of Pharmacy, Jagiellonian University Medical College, Medyczna 9, 30-688 Cracow, Poland; ∥ Department of Medicinal Chemistry, Faculty of Pharmacy, Jagiellonian University Medical College, Medyczna 9, 30-688 Cracow, Poland; ⊥ Selvita S.A., Bobrzyńskiego 14, 30-348 Cracow, Poland; # Department of Technology and Biotechnology of Drugs, Jagiellonian University Medical College, Medyczna 9, 30-688 Cracow, Poland; 7 Experimental Medicine Center, Medical University, Jaczewskiego 8, 20-090 Lublin, Poland; 8 Department of Molecular Biology, Institute of Biological Sciences, Maria Curie-Skłodowska University, Akademicka 19, 20-033 Lublin, Poland; 9 Department of Epizootiology and Clinic of Infectious Diseases, Faculty of Veterinary Medicine, University of Life Sciences in Lublin, Głęboka 30, 20-612 Lublin, Poland

**Keywords:** neuronal excitability, seizure disorders, antiseizure medications, drug discovery, neuroinflammation

## Abstract

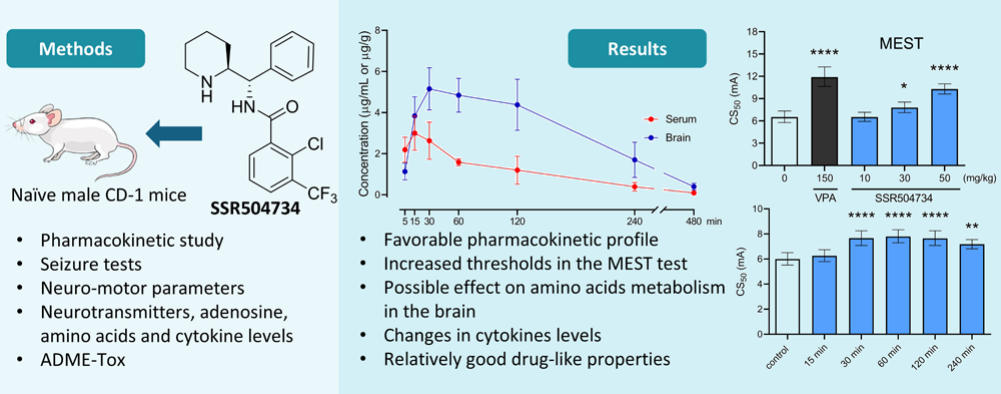

Studies have revealed that inhibition of glycine transporter type 1 (GlyT1) may provide a balanced regulation between excitation and inhibition in some brain structures and, thereby, modulate seizure activity. Data on the role of GlyT1 in epilepsy are, however, very limited. Here, we examined the effect of SSR504734, a highly selective and reversible GlyT1 inhibitor, on three acute seizure tests in mice. We also evaluated its impact on neurotransmitter levels in the relevant brain structures following seizures, possible adverse effects, and changes in the levels of inflammatory mediators in the serum and liver. In addition, in vivo pharmacokinetic profile and in vitro ADME-Tox properties of SSR504734 were investigated. The results show that SSR504734 significantly increased the threshold for tonic hindlimb extension in the MEST test after acute and repeated treatment but had no influence on seizure thresholds in the 6 Hz and i.v. PTZ seizure tests. SSR504734 did not affect the levels of glutamate, GABA, glycine, or adenosine in brain structures of mice with MES-induced seizures. However, after acute treatment, the concentration of glutamate and adenosine in the brainstem of control animals (i.e., without seizures) decreased. Moreover, SSR504734 increased the levels of inflammatory markers (TNF-α, Il-1β, IL-6, IL-10, and TLR4) in serum. In vivo pharmacokinetic profiling and in vitro ADME-Tox data confirmed suitable drug-like properties of SSR504734, including its notable penetration into brain tissue. However, possible hepatotoxicity at higher doses should be taken into account. Further studies should be considered to better characterize the SSR504734-mediated effects as well as to validate GlyT1 as a potential new molecular target in epilepsy treatment.

## Introduction

Epilepsy stands as one of the most common neurological central nervous system (CNS) diseases, impacting around 1% of the global population.^1,2^ It is characterized by an enduring tendency to generate spontaneous and recurring seizures due to excessive electrical discharges in the brain, leading to a variety of physical and behavioral symptoms.^2−5^ An imbalance between the inhibition and excitation processes in the CNS caused by changes in GABAergic and glutamatergic transmission, disruptions in the function of ion channels or pumps, and other factors are considered responsible for the development of seizures.^1^ Epilepsy treatment strategies aim to control seizures and improve patients’ quality of life, but in only 60–70% of epileptic patients, seizures can be controlled with antiseizure medications available on the market.^1,6,7^ The rest of the patients still experience constant, debilitating seizures. Therapies using several drugs may lead not only to undesirable interactions but also a number of side effects.^1,5,8^ Thus, further research is necessary to develop new, more effective, and innovative methods of treating epilepsy (including particularly the identification of new molecular targets for novel antiseizure medications).

Glycine is a fundamental amino acid, which plays a crucial role in metabolic processes (including biomolecule synthesis), immunomodulation, and antioxidation. It is also involved in neurotransmission at both inhibitory and excitatory synapses in the CNS and acts as a neuroprotective substance.^9,10^ Glycine works as an inhibitory neurotransmitter by acting as an agonist of the strychnine-sensitive glycine receptors (GlyRs) that are expressed predominantly in spinal cord, brainstem, and cerebellum.^11,12^ GlyRs are ionotropic ligand-gated channels of cysteine-loop receptors (Cys-loop), which mediate synaptic inhibitory neurotransmission and modulate neuronal excitability throughout the CNS via chloride ion influx and postsynaptic membrane hyperpolarization.^11,13,14^ They are involved in neurodevelopment, motor learning, motor and reflex activity, respiratory rates, muscle tones, and sensory processing.^11,15,16^ Glycine also works as a coagonist of the excitatory *N*-methyl-d-aspartate (NMDA) receptors that are widely distributed in the CNS.^12,17^ For activation, the NMDA receptor requires the binding of an endogenous agonist (l-glutamate) and an obligatory coagonist (glycine or d-serine).^18^ NMDA receptors play critical role in various brain functions.^19^

Extracellular glycine concentrations are controlled by two types of transporters—GlyT1 and GlyT2^20^ that belong to the family of sodium-/chloride-dependent solute carrier 6 family (SLC6) transporters. GlyT1 in the CNS is predominantly expressed in the cerebellum, brainstem, and spinal cord, with lower levels in the hippocampus and striatum. In the hindbrain, GlyT1 is primarily localized on glial cells where it modulates termination of inhibitory neurotransmission.^11,12,21−23^ It is also expressed on pre- and postsynaptic glutamatergic terminals, which can facilitate synaptic plasticity dependent on NMDA receptors by binding to the glycine_B_ site.^12^ Maintaining the concentration of extracellular glycine at an appropriate level is necessary for the stability of glycinergic and glutamatergic neurotransmission and dysregulation of glycine uptake promotes profound changes in the proper functioning of neurons and cognitive impairment.^21,24^ As a result, GlyT1 has emerged as a promising target for the treatment of various CNS diseases, including depression, anxiety, autism spectrum disorders, schizophrenia, neurodegenerative disorders, and potentially epilepsy.

Simultaneous activation of inhibitory GlyRs and excitatory NMDA receptors may provide a homeostatic regulation in some brain regions, for example, in the hippocampus that is highly implicated in epilepsy.^25^ Glycine can also regulate hippocampal inhibition through the GlyR-mediated downregulation of inhibitory GABA_A_ receptors. Moreover, hippocampal excitation may be regulated through the glycine binding site-dependent internalization of NMDA receptors. Consequently, by controlling extracellular glycine levels, GlyT1 may modulate the excitation/inhibition balance and thereby influence seizure susceptibility.^21,24,26^ However, the involvement of GlyT1 in epilepsy appears to be slightly under-appreciated in regard to both the pathophysiology of seizure/epilepsy and as the potential therapeutic target for seizure control.

Here, we aimed to provide more insight into the potential involvement of GlyT1 in control of seizure susceptibility by investigating the effect of SSR504734, a highly selective and reversible nonsarcosine GlyT1 inhibitor,^27^ in three models of acute seizures in mice after single and a 14-day administration. Furthermore, we evaluated the influence of SSR504734 on neurotransmitter (glycine, glutamate, GABA, and adenosine) concentrations in the hippocampus, brainstem, and cortex after seizures. To better characterize the tested compound, we assessed its in vivo pharmacokinetic profile, in vitro ADMET-Tox parameters such as permeability, plasma protein binding, metabolic stability, neurotoxicity, and hepatotoxicity, as well as the effect on the levels of inflammatory mediators in murine serum and liver. Possible adverse effects of SSR504734 on neuromuscular strength and motor coordination were also investigated.

## Materials and Methods

### Animals

All studies were performed on adult naïve male CD-1 mice (weighting 20–30 g at the beginning of the experiments) purchased from Charles River (Sulzfeld, Germany). Animals were adapted to the laboratory conditions for at least 1 week before the experiments. Mice were hosted in groups of 8–10 per cage at controlled environmental conditions (temperature 21–24 °C, relative humidity 45–65%, artificial 12 h light/dark cycle (light on at 6:00 a.m.), and free access to food pellets and tap water). The experiments were performed between 8:00 a.m. and 2:00 p.m. to minimize circadian influences with a minimum 30 min acclimatization period to the experimental room. Housing and experimental procedures were conducted under the guidelines provided by the European Union Directive of September 22, 2010 (2010/63/EU) and Polish acts concerning animal experimentation. All in vivo experimental procedures were approved by the Local Ethical Committee in Lublin (license nos. 38/2022 and 126/2022). Total number of used animals in this study was 782. Each mouse was used only once. In vivo experiments were performed by investigators who were blind to the treatment conditions.

### Treatment

SSR504734 (2-chloro-*N*-[(*S*)-phenyl[(2*S*)-piperidin-2-yl]methyl]-3-trifluoromethyl benzamide monohydrochloride) was synthesized at the Department of Medicinal Chemistry, Jagiellonian University (Cracow, Poland) according to the method described in detail in Supplementary file. SSR504734 and sodium valproate (VPA; Sigma-Aldrich) were dissolved in saline and injected intraperitoneally (i.p.). For pharmacokinetic profiling and a time-course study, SSR504734 was injected at several time points (15–240 min). In acute studies, SSR504734 and VPA (positive control) were administered 60 and 15 min before each test, respectively. The pretreatment time for SSR504734 was chosen based on the results from pharmacokinetic studies, the literature, and the experiment that evaluated the time-course of compound effect in the MEST test. In subchronic studies, SSR504734 and VPA were given repeatedly every 24 h for 14 consecutive days; the last injections were made 60 and 15 min before the tests, respectively. Negative control received vehicle only. All drug solutions were prepared freshly and administered at a volume of 10 mL/kg of body weight.

### Pharmacokinetic Study of SSR504734

SSR504734 was administered i.p. at the dose of 30 mg/kg, and mice were sacrificed by decapitation at 5, 15, 30, 60, 120, 240, and 480 min after dosing. The trunk blood samples (∼1 mL) were collected in polypropylene tubes and allowed to clot at room temperature. Subsequently, the blood was centrifuged at 5600 rpm for 10 min. Serum samples were collected in new tubes. Immediately after decapitation, brains and other tissues such as the liver, lungs, kidneys, heart, spleen, gut, and adipose were removed from the skull and washed with cold 0.9% NaCl. Samples were kept at −80 °C until the day of analysis.

Serum and tissue concentrations of SSR504734 in mice were quantified by a liquid chromatography tandem mass spectrometry (LC-MS/MS) method. Stock solutions of the studied compound were prepared in methanol at a concentration of 1 mg/mL. Working solutions were prepared by dilution of the standard solutions in acetonitrile/water 1:1 (v/v). Tissue homogenates were obtained by homogenization of each tissue in distilled water at a ratio of 1:4 (w/v) using a basic T10 ULTRA-TURRAX tissue homogenizer (IKA, Poland). Mouse serum and tissues, which were used to prepare standard samples and quality control (QC) samples, were harvested from untreated healthy animals after exsanguination. Standard and QC samples were prepared by adding 5 μL of working solutions of SSR504734 to 45 μL of mouse serum or 45 μL of tissue homogenates. Real serum and homogenate samples, QC samples, and standard samples were subjected to the same precipitation procedure. Briefly, 150 μL of 0.1% formic acid solution in acetonitrile containing pentoxifylline (PTX) as an internal standard (IS) at a concentration of 5 ng/mL was added to 50 μL of analyzed serum, homogenate, standard samples, or QC samples in Eppendorf tubes and mixed on a vortex mixer for 10 min (IKA Vibrax VXR, Germany). Subsequently, samples were centrifuged at 10,000 rpm for 10 min at room temperature (Eppendorf miniSpin centrifuge). 2 μL of supernatant was subjected to LC-MS/MS analysis using an Exion LC AC HPLC system coupled to a SCIEX QTRAP 4500 triple-quadrupole mass spectrometer (Danaher Corporation, Framingham, MA, US). Chromatographic separation was performed using a Hypersil Gold C18 analytical column (2.1 × 50 mm, 3 μm) (Thermo Scientific, USA) at 25 °C. The mobile phase consisting of water containing 0.1% formic acid and acetonitrile containing 0.1% formic acid (60:40, v/v) was pumped at a flow rate of 0.5 mL/min in an isocratic elution mode. All detections were performed in positive ion mode. The selected reaction monitoring (SRM) for the transitions was *m/z* 397.2 → 207.0 and *m/z* 397.2 → 174.2 (quantifier and qualifier) for SSR504734 and *m/z* 279 → 181 for IS. The optimal MS parameters for SSR504734 multiple reaction monitoring (MRM) transitions selected by continuous infusion of the standard solution at the rate of 7 μL/min using a Harvard infusion pump were as follows: the declustering potential was 120 V, the collision energy was 35.6 V, the entrance potential was 10 V, and the cell exit potential was 6 V. These parameters for IS were 60, 24, 10, and 6 V, respectively. An additional tuning optimization of gas flows and temperatures was performed by flow injection analysis. The ion source temperature was maintained at 450 °C, the ion spray voltage at 4000 V, the curtain gas (CUR) was set at 25 psi, the nebulizer (GS1) and TIS (GS2) gases at 40.0 and 50.0 psi, respectively, and the collision gas (CAD) at Medium.

Data acquisition and processing were carried out using Analyst 1.7 software. The calibration curves were constructed by plotting the ratio of the peak area of the analyte to the peak area of the IS vs the concentration of the analyte and generated by weighted (1/*y*) nonlinear regression analysis by fitting a quadratic formula. The validated quantitation ranges for SSR504734 were from 3.9 to 4000 ng/mL in serum, from 7.8 to 3000 ng/g in the brain, and from 0.2 to 50 μg/g in the remaining tissues. Samples with concentrations over the ULOQ were diluted with an appropriate matrix. The accuracy of samples was within 15% deviation from the nominal values for all standard samples and QCs except for the LLOQ, for which it was within 20%. The precision expressed as a relative standard deviation was within the acceptable limit of 15%. No significant matrix effect was observed, and there were no stability related problems during the routine analysis of the samples.

Pharmacokinetic parameters were calculated based on the concentration vs time data using noncompartmental analysis (PKanalix, Monolix suite, Lixoft, France).

### Intravenous (i.v.) PTZ Seizure Threshold Test

Mice were individually placed in a plastic restrainer (12 cm long, 3 cm inner diameter), and the lateral vein of each mouse was catheterized using a 27-gauge needle (Sterican, B. Braun Melsungen, Melsungen, Germany). The needle was attached by polyethylene tubing (PE20RW, Plastics One Inc., Roanoke, VA, USA) to a 10 mL plastic syringe containing 1% solution of PTZ in saline (Sigma-Aldrich, St. Louis, MO, USA) and mounted to a syringe pump (model Physio 22, Hugo Sachs Elektronik, Harvard Apparatus GmbH, March-Hugstetten, Germany). Piece of adhesive tape was used to secure the needle. The proper placement of the needle in the vein was verified by the appearance of blood in the tubing. During the test, mice were placed in a transparent box for observation. The PTZ solution was administered to freely moving mice at a constant rate of 0.2 mL/min. The time intervals from the initiation of PTZ infusion to the occurrence of the three following end points, i.e., (1) the first myoclonic twitch, (2) generalized clonic seizure with loss of righting reflex, and (3) forelimb tonus, were recorded. The PTZ infusion was terminated when tonic seizures, which were often fatal for the mice, began. All mice that survived were euthanized immediately. The thresholds were calculated separately for each end point using the following formula: threshold dose of PTZ (mg/kg) = infusion duration (s) × infusion rate (mL/s) × PTZ concentration (mg/mL)/body weight (kg). Seizure threshold was expressed as the amount of PTZ (mg/kg) ± SD (standard deviation) needed to produce the first observable sign of each end point. Each group consisted of 12–15 animals.

### Maximal Electroshock Seizure Test

The maximal electroshock seizures were induced through consistent current stimuli (50 Hz sinewave, 0.2 s) using a rodent shocker (type 221; Hugo Sachs Elektronik, Freiburg, Germany). A sinusoidal current alternation was delivered via saline-soaked transcorneal electrodes. Before stimulation, an ocular anesthetic (1% tetracaine hydrochloride solution obtained from Sigma-Aldrich, St. Louis, MO, USA) was administered to each eye of the mice. During stimulation, the mice were manually immobilized in a hand for 3–5 s and immediately relocated to a transparent box for behavioral observation after the stimulation. Hindlimb tonic extension was taken as an end point. Two experimental approaches were employed: (1) the maximal electroshock seizure threshold (MEST) test at varied current intensities and (2) the maximal electroshock (MES) test at a fixed current intensity.

In the MEST test, seizure thresholds were determined using the “up-and-down” method described by Kimball et al.^28^ Current intensities were adjusted in 0.06-log steps (from 5 to 13.2 mA), based on whether the previously stimulated animal did or did not exert tonic hindlimb extension, respectively. Each mouse was stimulated only once. Data obtained from groups of 20 animals were analyzed to establish the current threshold necessary to evoke the end point in 50% of the mice (CS_50_ with confidence limits for 95% probability).

In the MES test, animals were stimulated with supramaximal MES stimulus of 50 mA. Nonstimulated (sham) animals were treated identically to the MES stimulated mice except that no stimulus was delivered.

### 6 Hz-Induced Psychomotor Seizure Threshold Test

Psychomotor seizures were induced through the application of rectangular pulses (0.2 ms width; 6 pulses per second) for 3 s using a Grass model CCU1 constant current unit coupled to a Grass S48 stimulator (Grass Technologies, Warwick, RI, USA). Stimuli were delivered via saline-soaked transcorneal electrodes. An ocular anesthetic (1% tetracaine hydrochloride) was administered on the animal’s corneas. Mice were restrained manually during stimulation and placed in transparent box immediately for behavioral observation. 6 Hz-induced seizures were characterized by head-nodding, chewing, eye-blinking, twitching of the vibrissae, rearing, stunned posture, forelimb clonus, and Straub tail. The absence of the described features or the return to normal exploratory behavior within 20 s poststimulation was considered as the absence of seizures. The 6 Hz seizure threshold test were conducted on groups of 20 animals stimulated with different current intensities by using the “up-and-down” method described by Kimball et al.^28^

The current intensity was adjusted in 0.06-log intervals, either increased or decreased, depending on whether the previously stimulated animal exhibited a psychomotor seizure. The seizure threshold was quantified as the median current strength (CS_50_ value with confidence limits for 95% probability), predicting the induction of psychomotor seizures in 50% of the tested animals.

### Grip Strength Test

The effect of SSR504734 on neuromuscular strength was determined using the grip-strength apparatus (BioSeb, Chaville, France). The apparatus consists of a steel wire grid of 8 × 8 cm, connected to an isometric force transducer. Each mouse was held by the tail, allowing it to grasp the grid with its forepaws only and steadily pulled back until it released the grid. The maximum grip strength value (in newtons; N) was measured three times for each animal. The mean force was normalized to body weight and expressed in mN/g ± SD for each experimental group. In order to minimize the number of animals used, a grip-strength test was carried out shortly before seizure tests.

### Chimney Test

The impact of SSR504734 on motor coordination in mice was evaluated by using the chimney test. Each animal was individually placed inside a transparent Plexiglas tube with an inner diameter of 3 cm and a length of 30 cm. The tube was horizontally positioned on a table and shifted vertically once the mouse reached the opposite end. Within a 60 s time frame, the mouse was required to climb backward to escape from the tube. In order to minimize the number of animals used, a chimney test was carried out shortly before seizure tests.

### Determination of Neurotransmitters in Brain Structures

Mice were administered with SSR504734 or saline acutely or repeatedly for 14 days and subjected to maximal electroshock seizures 60 min after treatment. Immediately following seizure induction, the animals were decapitated. Brains were removed from the skull and washed with ice-cold 0.9% NaCl. Cortex, hippocampus, and brainstem were isolated by microdissection and collected into Eppendorf tubes. Samples were frozen in liquid nitrogen and kept at −80 °C until the day of analysis.

Adenosine, GABA, and glutamate concentrations in mouse cortex, brainstem, and hippocampus were determined using an LC-MS/MS method. Stock solutions of GABA, adenosine, glutamate, and their internal standards (ISs), i.e., adenosine-13C5, GABA-d6, and glutamate-d5 (Toronto Research Chemicals Inc., Canada), were prepared using methanol for GABA and its IS and deionized water for adenosine, glutamate, and their ISs and stored at 4 °C. The stock solutions of GABA, adenosine, and glutamate were then appropriately diluted with acetonitrile/water 1:1 (v/v) solution to achieve various concentration levels required for the experiments. Brain tissue samples were homogenized using a Bead Ruptor Elite, bead mill homogenizer (Omni International, USA) with 90 μL of deionized water per 10 mg of tissue. The resulting homogenates were further diluted at 1:10 to 1:100 (v/v) ratios (depending on the expected analyte concentration) with acetonitrile/water 1:1 (v/v) solution. Deproteinization and IS addition to the samples were then carried out by adding 80 μL of 0.1% formic acid (FA) solution in acetonitrile, spiked with the ISs at the final concentrations of 100 ng/mL for adenosine-13C5 and glutamate-d5, and 1 μg/mL for GABA-d6 to 20 μL of the samples, which were then vigorously shaken for 15 min (IKA Vibrax VXR, IKA Werke GmbH & Co. KG, Germany) and centrifuged for 5 min at 8000 rpm(Eppendorf miniSpin centrifuge, Eppendorf, Germany) to ensure a uniform dispersion of the ISs in each sample and complete protein removal. The supernatants were then placed in autosampler vials.

Analyte separation was performed on an Exion LC AC system (Sciex, USA) coupled with a QTRAP 4500 triple quadrupole MS instrument (Sciex, USA). A 0.2 to 2 μL aliquot of sample was injected via an autosampler maintained at 15 °C into an XBridge HILIC (Waters, Ireland) analytical column (2.1 × 150 mm, 3.5 μm) with the column oven set at 25 °C. The chromatographic separation was achieved at isocratic conditions using a mobile phase composed of 80% solvent A (0.1% FA in acetonitrile) and 20% solvent B (0.1% FA in deionized water) delivered at a flow rate of 0.4 mL/min. In these conditions, retention times of analytes and corresponding ISs were 2.4 min for adenosine and adenosine-13C5, 2.5 min for glutamate and glutamate-d5, and 2.2 min for GABA and GABA-d6.

MS detection employed electrospray ionization (ESI) in positive ion mode. According to the results of flow-injection analysis, curtain gas was set at 30 psi, ion spray voltage at 5500 V, and the source temperature at 450 °C, with ion spray gases 1 and 2 at 50 and 40 psi, respectively. MRM mode was used, targeting specific precursor-product ion transitions that were *m*/*z* 268 → 136 for adenosine, *m*/*z* 148 → 84 for glutamate, *m*/*z* 104 → 87 for GABA, *m/z* 273 → 136 for adenosine-13C5, *m*/*z* 110 → 93 for GABA-d6, and *m*/*z* 153 → 88 for glutamate-d5.

Data acquisition and processing were conducted with Analyst version 1.7 software. The calibration curves were constructed by plotting the ratio of the peak areas of the target analytes to those of their respective ISs against the concentrations of the analytes. These curves were derived using a weighted linear regression analysis method (1/*x*). Due to the availability of stable isotope-labeled standards and the high endogenous concentrations of adenosine, glutamate, and GABA in the brain tissue, the calibrators were prepared in water. The validated quantification ranges were determined to be between 10 and 1000 μg/g of brain tissue for GABA, achieving accuracy levels from 90.6 to 110.0%, between 10 and 2000 ng/g for adenosine with an accuracy of 89.2 to 112.0%, and between 100 and 2000 μg/g for glutamate with an accuracy of 87.8 to 108.2%. Throughout the routine sample analysis, no significant problems related to the stability or matrix effects were observed.

Glycine concentrations in brain structures were analyzed by using high-performance liquid chromatography with fluorescence detection (HPLC-FLD). A stock solution of glycine (20 mg/mL) was prepared in water and diluted with the same solvent to prepare working standard solutions at the concentration range of 0.2–20 μg/mL. The homogenates were obtained as described above, and they were centrifuged before analysis for 5 min at 10,000 rpm (Eppendorf miniSpin centrifuge). To prepare samples for analysis, 2 μL (for brainstem) or 5 μL (for the remaining structures) of homogenate supernatants was transferred to Eppendorf tubes and diluted with water to 20 μL. Then the samples or the same volumes of calibrators containing known amounts of glycine were combined with 5 μL of 0.1 M borate buffer solution (pH = 10.4) and 2 μL of *o*-phthaldialdehyde (OPA) working solution. The working solution was obtained by dissolving 2.2 mg of OPA in 50 μL of absolute ethanol, 50 μL of 1 M sodium sulfite, and 0.9 mL of 0.1 M borate buffer solution (pH = 10.4). The reaction mixture was vortexed for 15 s, spinned down to make sure that all the sample is at the bottom of the tube, and incubated for 3 min at room temperature in darkness. The aliquots of 5 μL were injected into the column. The HPLC system (Merck-Hitachi, Japan) consisted of a pump (model L-2130), an autosampler (model L-2200), and a fluorescence detector (model L-2485) operating at λ_ex._ = 220 and λ_em._ = 385 nm. The EZChrom Elite Client/Server v. 3.2 software was used for data collection and analysis. The chromatographic separation of glycine derivative was achieved on the Gemini NX C18 column, 250 × 4.6 mm ID (Phenomenex, USA), with 5 μm particles protected with a guard column filled with the same packing material. The mobile phase consisted of methanol (solvent A) and 0.05 M phosphate buffer adjusted to pH = 4.5 with phosphoric acid 30% (solvent B) and run in a gradient mode at a flow rate of 1 mL/min. The gradient program was set as follows: 15% of solvent A was decreased to 10% in 15 min, then it was increased to 42% in 1 min and kept constant for the next 4 min. The percentage of solvent A was then decreased to 32% in 1 min and kept constant until 23 min. Subsequently, it was reduced to 15% in 1 min and equilibrated at 15% until 27 min. The sample temperature in the autosampler vials was kept at 10 °C, and the column temperature was maintained at 35 °C throughout the whole analysis. The retention time of glycine was 6.3 min. There were no interfering peaks observed in the chromatograms at the retention time of the analyte. The calibration curve was constructed by plotting the peak areas versus known concentrations of glycine derivative in water. The method was specific, accurate (92–113%), and precise (CV% < 10). The OPA/sulfite derivative demonstrated appropriate stability upon storage in the autosampler over 24 h. The analytical run was much longer than the glycine derivative retention time to avoid the carry over in next run as other amino acid derivatives were eluted up to approximately 25 min under the conditions described above.

### Analysis of Inflammatory Markers in Serum and Liver

Samples of liver tissue were homogenized with a protease inhibitor solution (0.4 M NaCl, 0.05% Tween 20, 0.5% bovine serum albumin, 0.1 mM phenylmethylsulfonylfluoride, 0.1 mM benzethonium chloride, 10 mM EDTA, and 10 μg/mL aprotinin). Homogenates were centrifuged at 12,000 rpm at 4 °C, and supernatant fluids were collected and stored at −80 °C until analysis. The entire procedure was described by Zhao et al.^29^

ELISA kits were used to determine IL-1-β, IL-6, IL-10, TNF-α, and IFN-γ (EIAAB Science INC, Wuhan, Hubei, China, nos. E0563m, E0079m, E0056m, E0049m, E0133m, E0753m) and IL-18 (Biorbyt Ltd., Cambridge, United Kingdom, no. orb437211) in the liver homogenates and serum. All assays were performed according to the producer’s instructions. All samples were tested in triplicate.

### Binding Study

Binding of SSR504734 to GlyT1 was evaluated in Eurofins Panlabs Discovery Services Taiwan, Ltd. (New Taipei City, Taiwan) by use of the protocol described elsewhere.^30^ The experimental conditions are summarized in Table S1. The compound was dissolved in 1% DMSO and tested at concentrations of 0.1, 20, and 100 μM. Results are presented as the percent inhibition of specific binding (Table 2). All assays were carried out in duplicate.

### In Vitro ADME-Tox Assays

A series of in vitro ADME-Tox tests were performed for SSR504734 in order to estimate its drugability. The ADME-Tox parameters including permeability, plasma protein binding, metabolic stability, neurotoxicity, and hepatotoxicity were carried out as described previously.^31,32^ The ability of SSR504734 to penetrate through the biological membrane by passive diffusion was estimated by the Gentest Precoated PAMPA Plate System (Corning, Tewksbury, MA) and expressed as the permeability coefficient (Pe). The protein binding analysis was performed by using the commercial TRANSIL^XL^ PPB test, which mimics in vitro physiological plasma conditions where human serum albumin (HAS) and alpha-1-acid glycoprotein AGP are present in a 24:1 ratio. The human metabolism of SSR504734 was studied using human liver microsomes (HLMs) provided by Sigma-Aldrich. The hepatotoxicity and neurotoxicity were estimated in cellular models with the use of hepatoma HepG2 and neuroblastoma SH-SY5Y cell lines to perform the preliminary safety tests. The cells were incubated with SSR504734 at the concentration range of 0.1–100 μM. 1 μM cytostatic drug doxorubicin (DX) was used as the reference.

### Statistical Analysis

All data are presented as means ± SD unless otherwise stated. For statistical analysis of the data obtained from the MEST and the 6 Hz seizure threshold tests, the mean values of logarithms (of current strength) with standard deviations were used. These data were analyzed using a one-way analysis of variance (one-way ANOVA), followed by Dunnett’s post hoc test for multiple comparisons. Results from the chimney test were compared with Fisher’s exact probability. All other data sets were checked for normality with the Shapiro–Wilk test. Most of the data were normally distributed; therefore, parametric tests were used, i.e., one-way ANOVA (followed by Dunnett’s or Tukey’s post hoc test) or Student’s *t* test, where appropriate. Changes in neurotransmitter concentrations were analyzed using a two-way ANOVA with Tukey’s post hoc test. The factors of variation were SSR504734 treatment and seizures.

Statistical analyses were performed using GraphPad Prism 8 software for Windows (GraphPad Software, San Diego, CA, USA). A *p*-value of less than 0.05 was considered statistically significant.

## Results

### Pharmacokinetic Profile of SSR504734 in Mice

**Figure 1:**
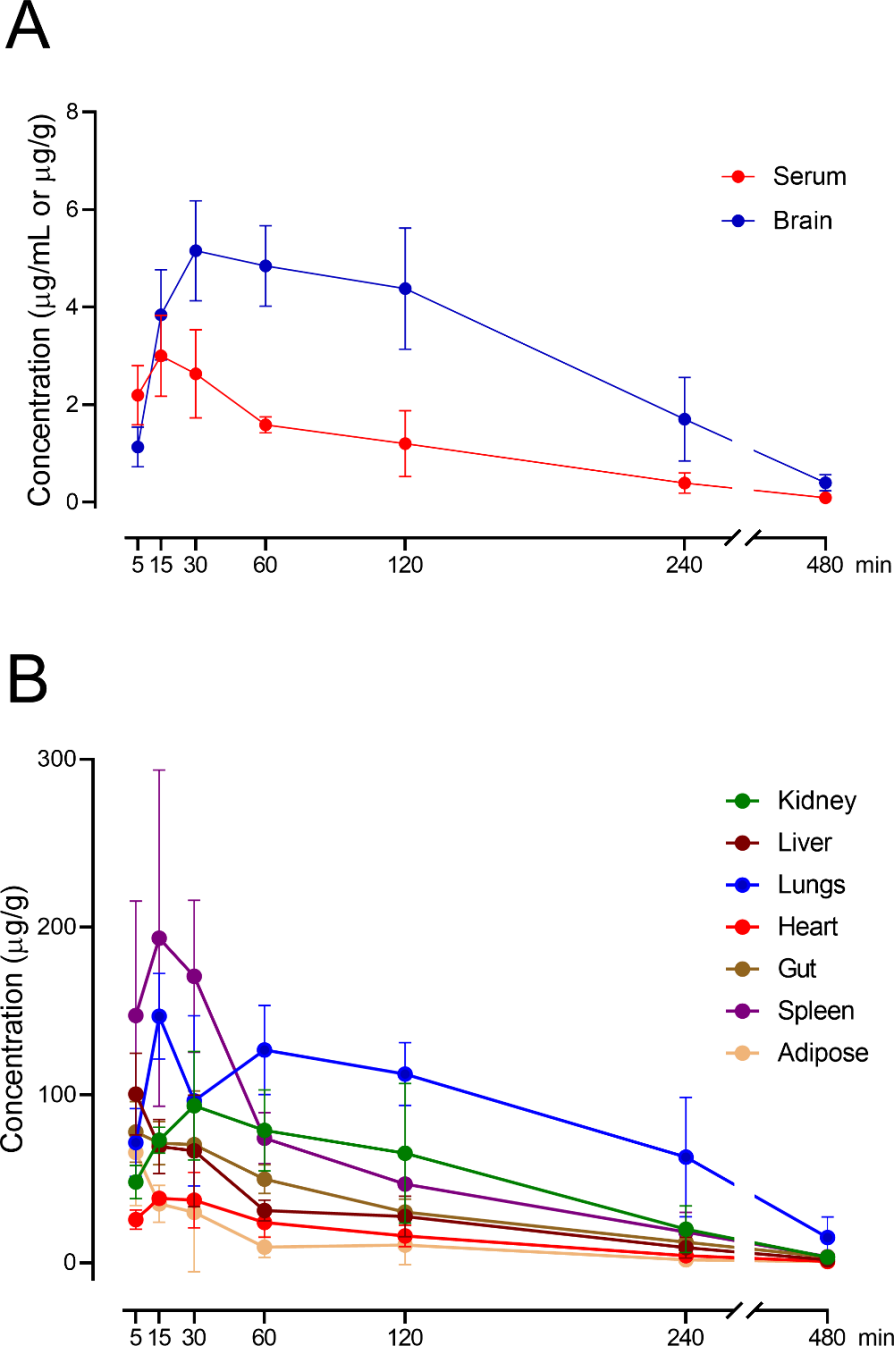
Concentration–time profiles of SSR504734 in the serum and brain (A) as well as different tissues (B) of mice given a single 30 mg/kg i.p. dose of the compound. Data are expressed as means ± SD (*n* = 4–6 animals/time point).

The pharmacokinetic evaluation of SSR504734 administered at an i.p. dose of 30 mg/kg in mice provides detailed information on the time course of its serum concentration and the extent of tissue distribution, including its levels attained at the site of action that are closely related to the therapeutic efficacy. A summary of pharmacokinetic parameters is presented in Table 1. From this table, the compound exhibited a tissue-to-serum partition coefficient (*K*_p_) of 3.05 in the brain, with the maximum concentration (*C*_max_) of 5.15 μg/g and a time to maximum concentration (*t*_max_) of 30 min. The terminal half-life (*t*_1/2_) of SSR504734 in this tissue considered the biophase of SSR504734 was 104.93 min and the mean residence time (MRT) was 141.31 min that reflects sustained exposure in the CNS, suggesting a high potential for effective GlyT1 inhibition. The total drug exposure in the brain, represented by the area under the concentration–time curve (AUC_last_), was 1137.50 min·μg/g, supporting the ability of SSR504734 to maintain therapeutic concentrations in the CNS (Figure 1A). Distribution across peripheral tissues revealed a high variability in both *C*_max_ and *K*_p_ values (Table 1 and Figure 1B). The compound exhibited the highest *K*_p_ and *C*_max_ in the lungs, followed by in the spleen and kidneys. The heart, liver, and gut also displayed a notable potential for accumulation of SSR504734, with *K*_p_ values of 12.99, 23.67, and 28.46, respectively. In addition, adipose tissue showed a moderate potential for accumulation of this compound, indicating that its sequestration in this tissue may occur.

**Table 1:** Summary of Pharmacokinetic Parameters of SSR504734 in Different Tissues of Mice Given a Single 30 mg/kg i.p. Dose of This Compound^a^

	*t*_1/2_ (min)	*C*_max_ (μg/mL (g tissue))	*t*_max_ (min)	MRT (min)	AUC_last_ (min·μg/mL (g tissue))	*K*_p_
serum	99.15	3.00	15	111.61	372.73	
brain	104.93	5.15	30	141.31	1137.50	3.05
heart	78.73	38.27	15	101.00	4841.83	12.99
lungs	122.45	146.83	15	156.22	33485.57	89.84
kidneys	84.08	93.52	30	121.82	16781.00	45.02
liver	91.35	100.54	5	105.50	8821.36	23.67
spleen	96.04	193.42	15	99.55	18616.26	49.95
gut	103.89	77.92	5	117.53	10606.43	28.46
adipose	79.63	65.70	5	82.17	3347.89	8.98

a *t*_1/2_—terminal half-life, *C*_max_—maximum concentration of the compound, *t*_max_—time to maximum concentration, MRT—mean residence time, AUC_last_—area under the concentration−time curve to the last measured point, *K*_p_—tissue-to-serum partition coefficient (*K*_p_ = AUC_last(tissue)_/AUC_last(serum)_).

Rapid distribution was observed to most tissues, with an *t*_max_ of 15 min in the heart, lungs, and spleen. The liver exhibited the shortest *t*_max_ of SSR504734 equal to 5 min, which is consistent with its role in early drug metabolism and the i.p. route of administration. The total drug exposure was the highest in the lungs followed by the spleen and kidneys, suggesting these organs may serve as primary reservoirs for SSR504734. The apparent clearance (CL/F) and volume of distribution during the terminal phase (Vz/F) calculated based on the serum vs time profile for the tested compound were 0.0778 L/min/kg and 11.13 L/kg, respectively. The latter value confirms the extensive tissue distribution of SSR504734 in mice, as Vz/F is much larger than that of the mouse body water.

### Time-Course of SSR504734 Effect in the MEST Test

**Figure 2:**
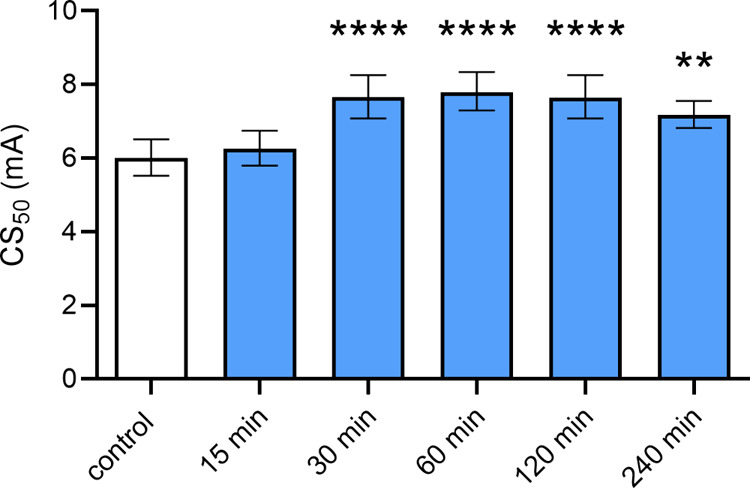
Time-course effect of SSR504734 in the MEST test in mice. SSR504734 (30 mg/kg) was administered i.p. 15, 30, 60, 120, and 240 min prior to the test. Control animals received saline. Results are presented as median current strengths (CS_50_ in mA with their 95% confidence limits) required to produce tonic hindlimb extension in 50% of animal tested. Each group consisted of 20 animals. Statistical analysis: **p < 0.01, ****p < 0.0001 vs the control group (one-way ANOVA with Dunnett’s post hoc test).

As shown in Figure 2, SSR504734 (30 mg/kg) significantly raised the seizure threshold after i.p. administration at 30, 60, 120, and 240 min in the MEST in mice (one-way ANOVA, *F*(5,52) = 1.00; *p* < 0.0001). The highest effect (∼1.3-fold increase) was observed 60 and 120 min after administration (*p* < 0.0001 vs control group).

### Effect of Acute and 14-Day Treatment with SSR504734 on Seizure Threshold in the MEST Test

**Figure 3:**
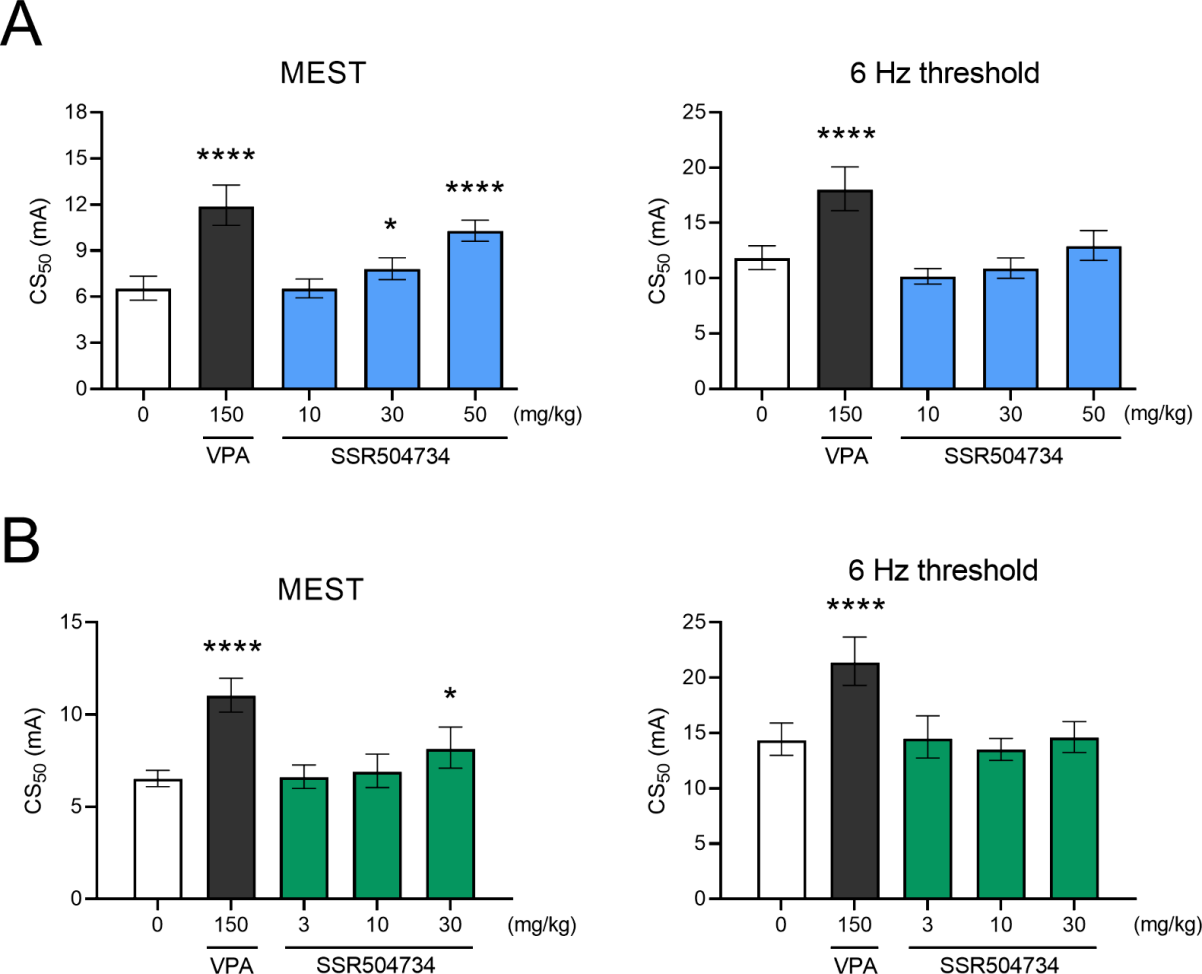
Effect of (A) acute and (B) 14-day treatment with SSR504734 on seizure thresholds in the MEST and 6 Hz tests in mice. In acute studies, SSR504734 and sodium valproate (VPA; positive control) were injected i.p. 60 and 15 min before the test, respectively. In subchronic studies, SSR504734 and VPA were injected i.p. every 24 h for 14 days. The last administration was made 60 and 15 min before the tests, respectively. Control animals received saline. Each experimental group consisted of 20 animals. Data are expressed as CS_50_ (in mA) values with 95% confidence limits. Each CS_50_ value represents the current intensity predicted to produce seizures in 50% of the mice. Statistical analysis: *p < 0.05, ****p < 0.0001 vs the control group (one-way ANOVA followed by Dunnett’s post hoc test).

The influence of SSR504734 on the threshold for the tonic hindlimb extension in the MEST test after single (one-way ANOVA, *F*(4,43)=29.71; *p* < 0.0001) and 14-day administration (one-way ANOVA, *F*(4,45)=17.11; *p* < 0.0001) is shown in Figure 3A,B. SSR504734 administered acutely at the dose of 10 mg/kg was ineffective. At higher doses of 30 and 50 mg/kg, it significantly increased current intensity necessary to induce hindlimb tonus (1.2-fold increase, *p* < 0.05, and 1.6-fold increase, *p* < 0.0001, respectively). After repeated treatment, SSR504734 at 30 mg/kg caused a 1.2-fold increase in CC_50_ value (*p* < 0.05). At lower doses (3 and 10 mg/kg), no changes in seizure threshold were found. For comparison, positive control (VPA, 150 mg/kg) significantly increased the seizure threshold in the MEST test after single (1.8-fold, *p* < 0.0001) and 14-day administration (1.7-fold, *p* < 0.0001).

### Effect of Acute and 14-Day Treatment with SSR504734 on Seizure Threshold in the 6 Hz-Induced Seizure Test

SSR504734 at any of the doses tested had no statistically significant effect on the current intensity necessary to induce psychomotor seizures after single (one-way ANOVA, *F*(4,41)=17.16; *p* < 0.0001; Figure 3A) and 14-day administration (one-way ANOVA, *F*(4,38) = 10.57; *p* < 0.0001; Figure 3B). By contrast, VPA at 150 mg/kg caused a marked increase of the threshold for the 6 Hz-induced seizures after both acute and 14-day treatment (*p* < 0.0001).

### Effect of Acute and 14-Day Treatment with SSR504734 on Seizure Threshold in the i.v. PTZ Test

**Figure 4:**
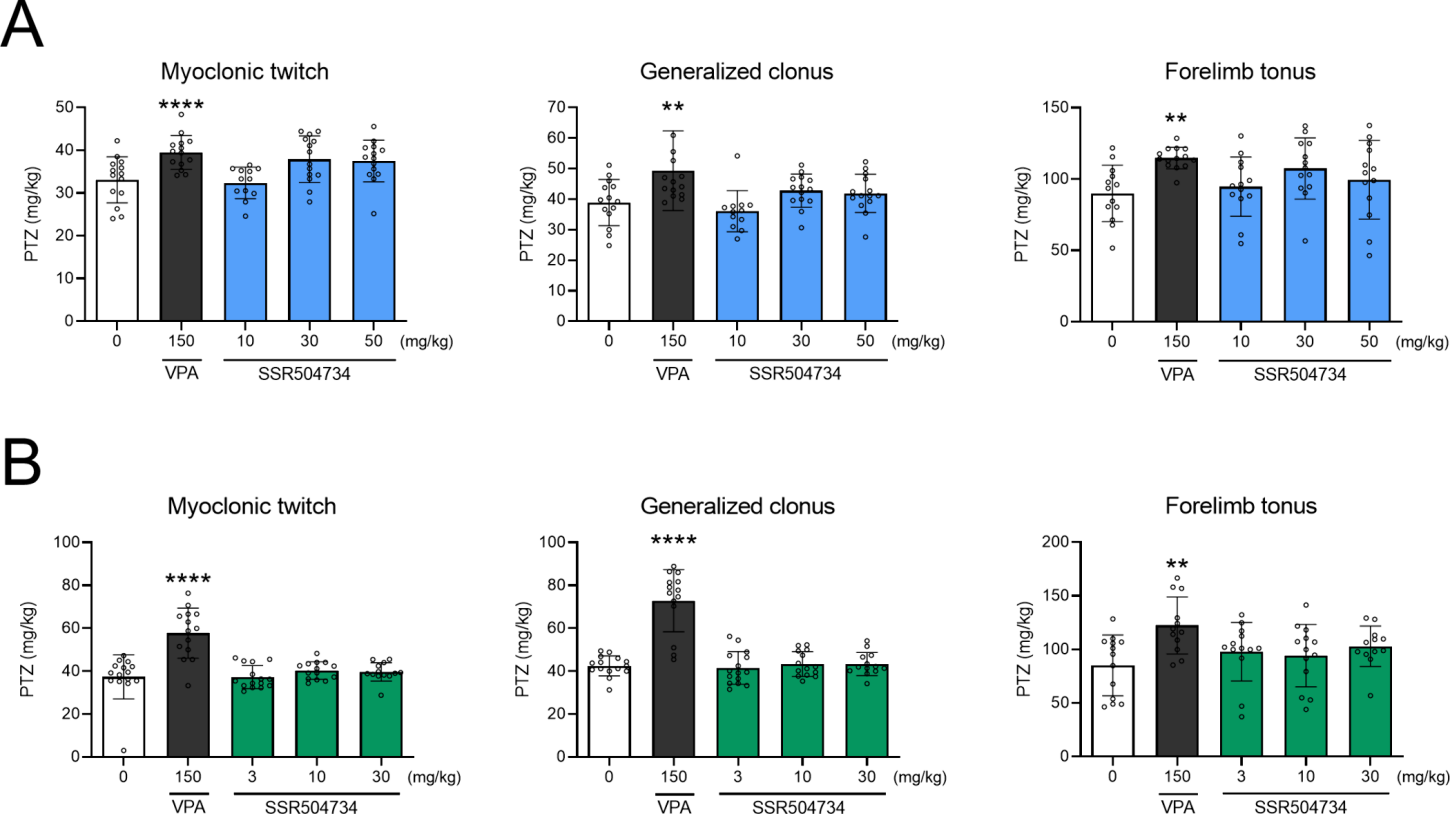
Effect of (A) acute and (B) 14-day treatment with SSR504734 on the threshold for the onset of the first myoclonic twitch, generalized clonus, and forelimb tonus in the i.v. PTZ test in mice. In acute studies, SSR504734 and sodium valproate (VPA; positive control) were injected i.p. 60 and 15 min before the test, respectively. In subchronic studies, SSR504734 and VPA were injected i.p. every 24 h for 14 days. The last administration was made 60 and 15 min before the tests, respectively. Control animals received saline. Data are expressed as means (mg/kg PTZ) ± SD, *n* = 12–15. Statistical analysis: **p < 0.01, ****p < 0.0001 vs the control group (one-way ANOVA followed by Dunnett’s post hoc test).

SSR504734 had no significant effect on the i.v. PTZ-induced seizures after single (one-way ANOVA: *F*(4,63) = 5.80; *p* = 0.0005 for myoclonic twitch; *F*(4,62) = 4.97, *p* = 0.002 for generalized tonus; *F*(4,61) = 3.18; *p* = 0.02 for forelimb tonus; Figure 4A) and 14-day treatment (one-way ANOVA: *F*(4,66)=17.00; *p* < 0.0001 for myoclonic twitch; *F*(4,66) = 36.58; *p* < 0.0001 for generalized clonus; *F*(4,58) = 3.41; *p* < 0.014 for forelimb tonus; Figure 4B). VPA at a dose of 150 mg/kg significantly increased the thresholds for all of the studied end points after single (*p* < 0.0001 for myoclonic twitch; *p* < 0.01 for generalized clonus and forelimb tonus) and repeated administration (*p* < 0.0001 for myoclonic twitch and generalized clonus; *p* < 0.01 and forelimb tonus).

### Effect of SSR504734 on Muscular Strength and Motor Coordination

In a time-course study, SSR504734 (30 mg/kg) significantly decreased neuromuscular strength at 15 min post administration (*p* < 0.05). No changes in the grip-strengths at 30, 60, 120, and 240 min post injection were reported (one-way ANOVA: *F*(5,66) = 2.44; *p* = 0.04). In the chimney test, SSR504734 did not affect motor coordination (Fisher’s exact probability test: *p* > 0.05) at any of the studied time points (Table S2).

In dose–response studies, acute administration of SSR504734 at 10, 30, and 50 mg/kg did not produce significant changes in muscle strength (one-way ANOVA: *F*(4,55) = 0.27; *p* = 0.90) and motor coordination (Table S3). It is, however, noteworthy that at the highest dose tested (i.e., 50 mg/kg), it caused motor impairment in 33.3% of animals (*p* = 0.09). To avoid neurotoxicity, lower doses were tested in a subchronic experiment. No alterations in the neuromuscular strength (one-way ANOVA: *F*(4,55) = 1.78; *p* = 0.15) and motor coordination were observed after 14-day administration of SSR504374 at doses of 3, 10, and 30 mg/kg (Table S3).

### Effect of Acute and 14-Day Treatment with SSR504734 on Neurotransmitter Levels after MES-Induced Seizures

**Figure 5:**
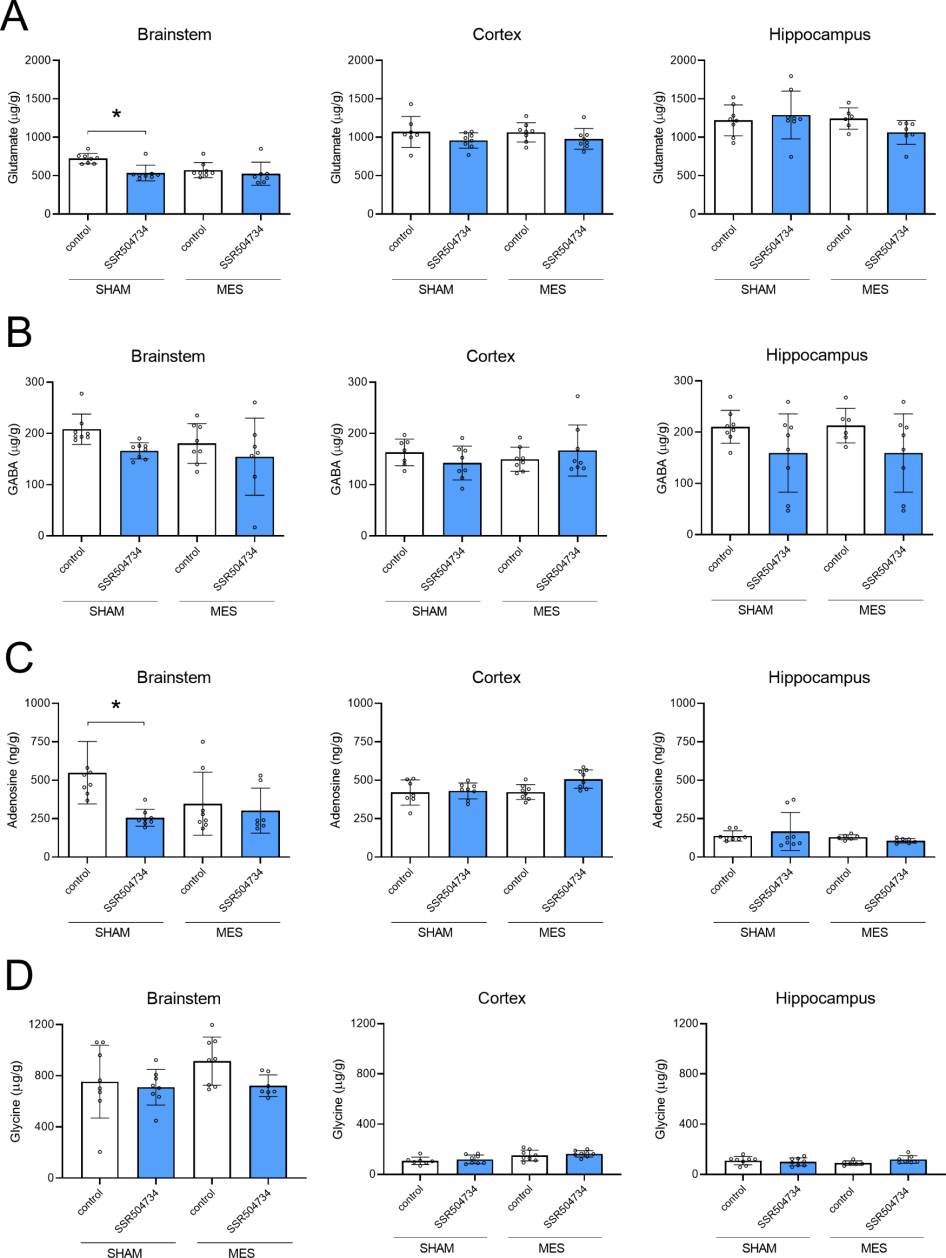
Effect of acute treatment with SSR504734 on (A) glutamate, (B) GABA, (C) adenosine, and (D) glycine concentrations in brain structures. SSR504734 was injected i.p. 60 min before seizure induction (MES). Control animals received saline. Nonstimulated (sham) animals were treated with saline or SSR504734, but they did not receive MES stimulus. Data are expressed as means ± SD. Statistical analysis: *p < 0.05 (two-way ANOVA followed by Bonferroni’s post hoc test).

The effect of acute treatment with SSR504734 on neurotransmitter levels in brain structures of nonstimulated (sham) and MES-stimulated mice is shown in Figure 5. A two-way ANOVA revealed no interaction between treatment with SSR504734 and seizures (*F*(1,27) = 3.22, *p* = 0.084), but a significant effect of treatment (*F*(1,27) = 9.33, *p* = 0.005) and a significant effect of seizures (*F*(1,27) = 4.31, *p* = 0.048) on the glutamate level in the brainstem. Bonferroni’s post hoc test showed that SSR504734 caused a significant decrease of glutamate concentration in nonstimulated mice (*p* < 0.05) with no statistically significant changes in mice subjected to the MES test. No alterations in glutamate concentration in cortex and hippocampus were reported. A two-way ANOVA also revealed a significant interaction between acute treatment with SSR504734 and seizures (*F*(1,27) = 4.38, *p* = 0.046), a significant effect of treatment (*F*(1,27) = 8.10, *p* = 0.008), and no significant effect of seizures (*F*(1,27) = 1.72, *p* = 0.200) on adenosine concentrations in the brainstem. A post hoc analysis showed a significant decrease of adenosine concentration following SSR504734 injection only in nonstimulated mice (*p* < 0.05). It is noteworthy that SSR504734 caused an ∼2-fold decrease in concentration of this neuromodulator. There were no changes in adenosine concentrations in cortex and hippocampus. Likewise, acute treatment with SSR504734 did not alter GABA and glycine concentrations in any of the studied brain regions.

The influence of a 14-day treatment with SSR504734 on neurotransmitter levels in brain structures of nonstimulated (sham) and MES-stimulated mice is shown in Figure S1. No alterations in glutamate, GABA, adenosine, or glycine concentrations were found. Statistical details for all comparisons are provided in the Supporting Information (Tables S4 and S5).

### Effect of 14-Day Treatment with SSR504734 on Inflammatory Markers in Serum and Liver

**Figure 6:**
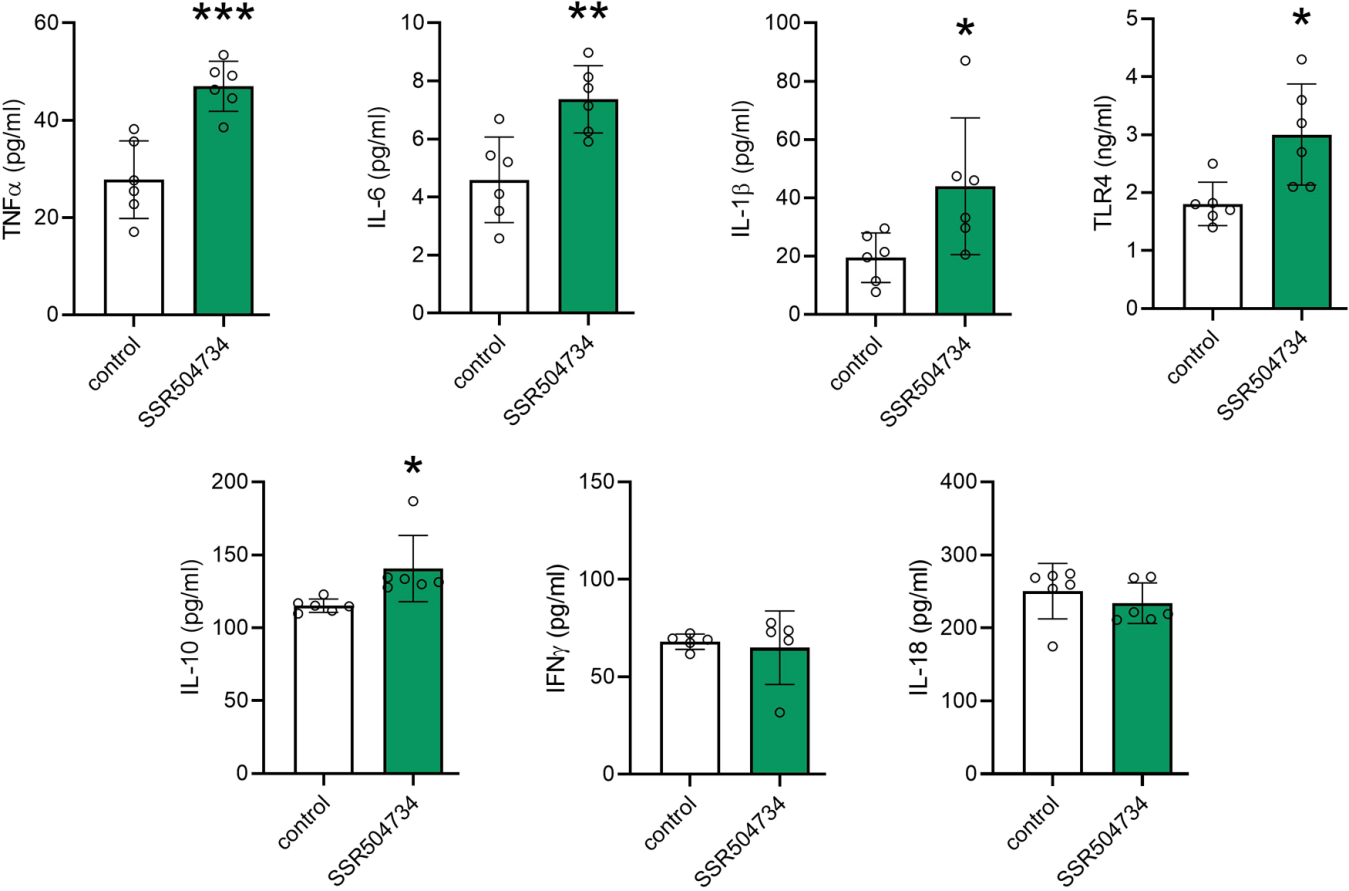
Effect of 14-day treatment with SSR504734 on inflammatory markers in serum. SSR504734 was injected i.p. every 24 h for 14 days. Control animals received saline. Data are expressed as means ± SD, *n* = 5–6/group. Statistical analysis: *p < 0.05, **p < 0.01, ***p < 0.001 (unpaired Student’s *t* test).

Changes in cytokine concentrations in serum after SSR504734 treatment are listed in Figure 6. A 14-day administration of SSR504734 at the dose of 30 mg/kg caused a significant increase in serum concentration of TNFα (*t* = 4.96; df = 10; *p* < 0.001), IL-6 (*t* = 3.62; df = 10; *p* = 0.005), IL-1β (*t* = 2.41; df = 10; *p* = 0.04), TRL4 (*t* = 3.09; df = 10; *p* = 0.01), and IL-10 (*t* = 2.69; df = 10; *p* = 0.02). There were no statistically significant differences in serum concentrations of IFN-γ (*t* = 0.36; df = 8; *p* = 0.73) and IL-18 (*t* = 0.86; df = 10; *p* = 0.41). No statistically significant changes in the level of inflammatory markers were revealed in liver samples (Figure S2 and Table S6).

### Binding

The binding results of SSR504734 to rat GlyT1 revealed a moderate interaction of compound at a concentration of 0.1 μM and a significant effect at higher concentrations of 20 and 100 μM (Table 2). The IC_50_ value for the reference GlyT1 inhibitor sarcosine in this testing system was 55.8 μM, suggesting a more potent interaction of SSR504734 with GlyT1 (52% inhibition observed at 20 μM).

**Table 2:** GlyT1 Binding Data for SSR504734 in the Rat Brain Cortex

concentration [μM]	% inhibition of control specific binding^a^
0.1	42
20	52
100	62

a Results showing activity higher than 50% are considered to represent significant effects of the test compounds; results showing an inhibition between 25 and 50% are indicative of a moderate effect.

### In Vitro ADME-Tox Assays

SSR504734 was tested in the parallel artificial membrane permeability assay (PAMPA) that included well-permeable caffeine as a reference. As shown in Table 3, SSR504734 exhibited an excellent permeability coefficient (*Pe* = 14.80 × 10^–6^ cm/s) that was almost double value of the used well permeable reference caffeine (*Pe* = 7.77 × 10^–6^ cm/s).

**Table 3:** ADMET Parameters of SSR504734 Determined In Vitro

	PAMPA	PPB	PPB			
compound	*Pe* [10^–6^ ± SD cm/s]	*K*_D_ [μM]	*f*_b_ [% ± SD]	metabolic stability^a^ [% remaining]	neurotoxicity [IC_50_, μM]	hepatotoxicity [IC_50_, μM]
SSR504734	14.80 ± 1.49	8.04	98.7 ± 0.13	94.72	38.05	14.75
reference drugs	caffeine	warfarin	warfarin	verapamil	doxorubicin	doxorubicin
	7.77 ± 2.30	9.50	98.5 ± 2.10	30.84	0.30	1.91

a After 120 min of incubation with human liver microsomes.

Determined in the plasma protein binding test, the dissociation constant of SSR504734 (*k*_D_) was 8.04 μM, which corresponds to 98.7% binding to plasma proteins. The results indicated that SSR504734 is highly bound to plasma proteins, similar to the positive control warfarin used (Table 3).

**Figure 7:**
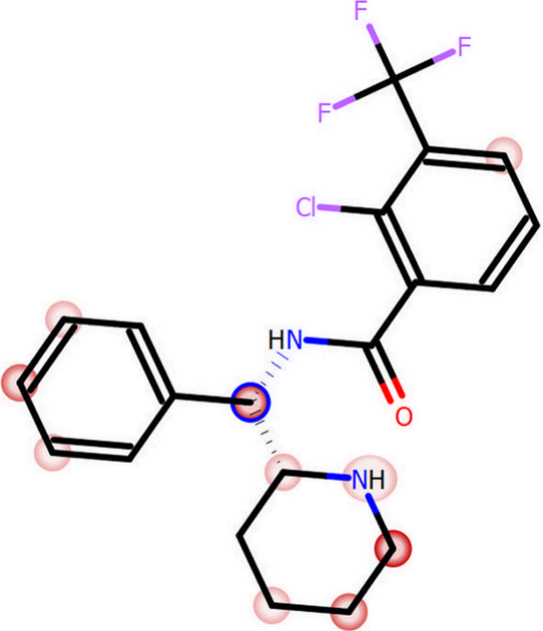
MetaSite 6.0.1. software prediction of the most probable sites of SSR504734 metabolism. The darker red color, the higher probability to be involved in the metabolism pathway. The blue circle marked the site of the compound with the highest probability of metabolic bioconversion.

The metabolic stability of SSR504734 was determined in silico and in vitro. The MetaSite 6.0.1 software predicted the most probable sites of tested compound metabolism (Figure 7) and metabolic pathways (data not shown). The MetaSite 6.0.1 suggests that the carbon atom connecting the benzyl and piperidine rings in the SSR504734 structure is the most susceptible for metabolism. Moreover, piperidine itself can also be considered as the site of the potential biotransformation.

The incubation with human liver microsomes (HLMs) (Table 3) for 120 min and further UPLC analysis (Figure S3) of the reaction mixture indicated that SSR504734 was metabolized only in 5%. This is an excellent result if we compare it to the metabolically unstable drug verapamil metabolized in 70% yield under the same conditions.

The UPLC analysis also showed the presence of three metabolites. The MS spectra are supported by the MetaSite 6.0.1. software that allowed us to identify the metabolic pathways (Table 4). The most probable structures of metabolites are shown in Figures S5–S7.

**Table 4:** Metabolic Pathways Summary: Molecular Masses and Metabolic Pathways of SSR504734 and Verapamil (Reference Unstable Drug) after Incubation with Human Liver Microsomes (HLMs)

substrate	molecular mass (*m*/*z*)	molecular mass of the metabolite (*m*/*z*)	metabolic pathway
SSR504734	397.21	425.15 (M1)	hydroxylation, oxidation, and dehydrogenation
		395.08 (M2)	dehydrogenation
		410.98 (M3)	oxidation
Verapamil	455.42	441.35 (M1)	demethylation
		291.33 (M2)	decomposition
		165.09 (M3)	decomposition
		441.29 (M4)	demethylation
		427.33 (M5)	double-demethylation
		277.26 (M6)	decomposition

**Figure 8:**
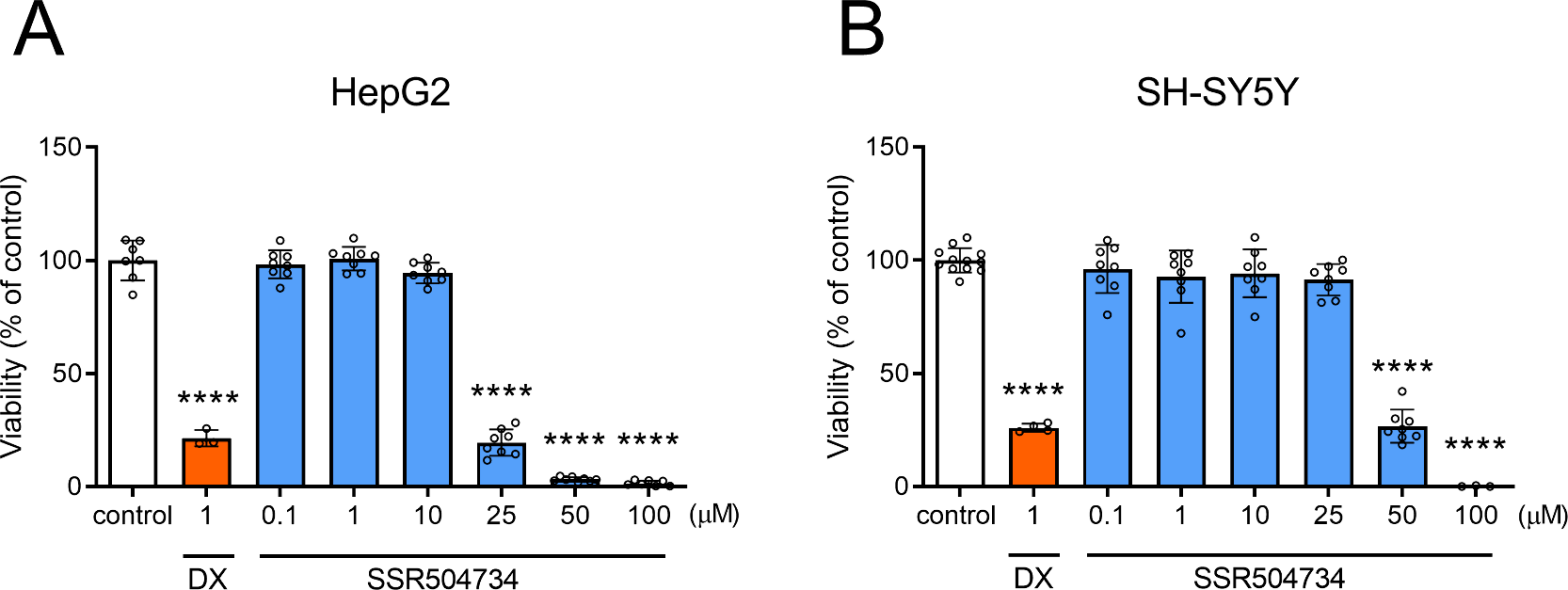
Effect of cytostatic drug doxorubicin (DX) and SSR504734 on (A) hepatoma HepG2 and (B) neuroblastoma SH-SY5Y cell lines viability after 72 h of incubation at 37 °C, 5% CO_2_. Data are expressed as means ± SD. Statistical analysis: ****p < 0.0001 vs negative control −1% DMSO in growth media (one-way ANOVA, followed by Dunnett’s post hoc test).

To determine the safety profile of SSR504734, its hepatotoxicity and neurotoxicity effects in HepG2 and SH-SY5Y cellular models were investigated. As shown in Figure 18, the tested compound did not exhibit hepatotoxic properties in the concentration range of 1–10 μM, whereas doxorubicin (a reference cytostatic drug) was highly toxic at 1 μM. However, the potential hepatotoxic risk of SSR504734 in vivo should be considered, as the significant decrease of cells’ viability was observed at 25 μM and higher concentrations. On the other hand, SSR504734 showed a weaker toxic effect in the SH-SY5Y model, with a completely safe dose of 25 μM and a decrease in cell viability to around 30% at 50 μM (Figures 8 and S8).

## Discussion

Inhibition of GlyT1 has gained considerable interest from the medical community and the pharmaceutical industry as one of the strategies in the development of novel therapies for various central nervous system disorders.^11,20,33,34^ GlyT1 may provide a balanced regulation between excitation and inhibition in some brain structures, and thereby, it may also modulate seizure activity. However, data on the role of GlyT1 in seizures and epilepsy are very limited. To date, it was found that GlyT1 is pathologically overexpressed in human patients with temporal lobe epilepsy and in epileptic animals,^24^ while pharmacological inhibition or genetic deletion of GlyT1 may produce antiseizure-like effects in rodents.^24,35−38^

Sarcosine, a naturally occurring amino acid belonging to the family of *N*-methylated derivatives of glycine, is one of the most widely investigated GlyT1 inhibitors.^39^ Its antiseizure-like properties were described in several studies. Sarcosine reduced seizure frequency and death in the strychnine-induced seizure test,^40^ delayed the onset and decreased duration of PTZ-induced seizure in rats,^41^ and raised the threshold for tonic hindlimb extension in the MEST test in mice.^37^ In the rapid kindling model in rats, sarcosine produced anti-ictogenic and antiepileptogenic effects.^36^ Additionally, it reduced the overexpression of hippocampal GlyT1 in kindled rats.^36^ While these findings are encouraging, it is important to note that sarcosine is actually a very weak competitive inhibitor of GlyT1 (IC_50_ = 91 μM). It likely has poor penetration through the blood–brain barrier, and relatively high doses are needed for in vivo effects.^34,42,43^ Kalinichev et al.^35^ described the effect of six more potent, selective, and chemically distinct GlyT1 inhibitors (NFPS, SSR504734, LuAA21279, Org25935, SB-710622, and GSK931145) on the seizure threshold in the MEST test in rats. All of them caused a strong, dose-dependent increase in the threshold for tonic hindlimb extension. Another GlyT1 inhibitor, LY2365109, increased seizure thresholds in the MEST- and PTZ-induced seizure tests and suppressed chronic seizures in the kainic acid-induced model of temporal lobe epilepsy in mice, but at higher doses, it triggered lethal respiratory arrest.^24^

SSR504734 is a potent, selective (vs 120 different transporters, receptors, ion channels, or enzymes), and reversible GlyT1 inhibitor. In the study by Depoortère et al.,^27^ it increased extracellular levels of glycine in the rat prefrontal cortex, potentiated NMDA-mediated excitatory postsynaptic currents in rat hippocampal slices, exhibited activity in animal models of schizophrenia, anxiety, and depression, as well as enhanced working memory performance. All of these make it a compelling candidate for further evaluation in seizure models. Epilepsy is a heterogeneous disease with complex pathophysiology, and different types of seizures may involve different mechanisms and/or brain areas.^2^ Therefore, we decide to use three different seizure models to evaluate the influence of SSR504734 after acute and 14 day treatments on different types of seizures. We found that SSR504734 significantly increased the seizure threshold for tonic hindlimb extension in the MEST test after both acute and repeated treatment, which is consistent with the results obtained by Kalinichev et al.^35^ This model is considered analogous to generalized tonic-clonic seizures in humans, and it is useful for identifying compounds that work as sodium channel blockers.^44^ In the i.v. PTZ seizure threshold test, which is the most sensitive method for measuring seizure thresholds in rodents, SSR504734 had no impact on any of the studied endpoints, i.e., first myoclonic twitch, generalized clonus, and tonic forelimb extension. SSR504734 also failed to affect the threshold for the 6 Hz-induced psychomotor seizures, which serve as a model of focal (limbic) seizures occurring in human partial epilepsy. A possible explanation for the differential effect of SSR504734 on seizure susceptibility could be the fact that different areas of the brain are involved in different types of seizures. Tonic seizures originate from the brainstem, whereas clonic seizures originate from the forebrain. These structures have distinct mechanisms for seizure generation and different thresholds for seizures initiation.^45,46^ It is suggested that electroshock directly affects the neural mechanisms in the brainstem responsible for triggering tonic hindlimb extension in rodents.^45^ GlyT1 and GlyR are expressed predominantly in those brain regions where glycine works as an inhibitory neurotransmitter, i.e., in the brainstem and spinal cord. Thus, by inhibiting GlyT1 and increasing the glycine concentration, SSR504734 may potentiate inhibitory GlyR-mediated neurotransmission in the brainstem and raise the threshold for tonic hindlimb extension. Since glycinergic neurons are less widely distributed in the forebrain, SSR504734 does not affect the thresholds for seizures originating in forebrain structures. It is noteworthy that similar findings were obtained for compound M22, which is another highly selective GlyT1 inhibitor. Although M22 significantly elevated seizure threshold in the MEST test in mice, it had no influence on the i.v. PTZ-induced seizures.^38^ Likewise, sarcosine increased the threshold for tonic seizure in the MEST test and was devoid of any effect in the i.v. PTZ test.^37^ In other studies, sarcosine did, however, affect seizures of the forebrain origin. But, as mentioned above, sarcosine is not an ideal tool to study the GlyT1-mediated effects as it is a weak GlyT1 inhibitor with various off-target effects, e.g., it also works as a coagonist of NMDA receptors^47^ and an agonist of GlyRs.^48^

The potential adverse effects of SSR504734 on neuromuscular strength and motor coordination were investigated in conjunction with seizure threshold tests. In a time-course study, we noted a slight decrease of neuromuscular strength at 15 min after i.p. administration of SSR504734 at 30 mg/kg. The effect was short-term, as no changes were observed at 30–240 min post administration. In a dose–response study, acute injection of SSR504734 at the highest dose tested of 50 mg/kg did not affect the neuromuscular strength, but it caused an impairment of motor coordination in ∼33% of animals tested. Though the changes were not statistically significant, we decided to use lower doses of SSR504734 in subchronic studies to avoid potential neurotoxicity after repeated treatment. The effects of SSR504734 on neuromuscular strength and motor coordination likely result from its impact on glycinergic neurons, mostly expressed in the brainstem and spinal cord, where they control various motor functions.

To provide more mechanistic insights into the effect of SSR504734 on tonic seizures, we determined concentrations of glutamate, GABA, glycine, and adenosine, all highly implicated in seizure activity, in brain structures of MES-stimulated mice, i.e., following tonic hindlimb extension. No alterations were found in MES-stimulated mice after both acute and repeated treatments with SSR504734. It should be noted here that neurotransmitter concentrations were measured in tissue homogenates and not in dialysates, which would be a more adequate approach. Interestingly, SSR504734 following acute, but not repeated, administration significantly decreased the concentrations of glutamate and adenosine in the brainstem of nonstimulated (sham) animals. An unexpected decrease in glutamate in the brainstem may reflect the imbalance in the endogenous amino acid pool induced by GlyT1 inhibition. Inhibition of GlyT1 by SSR504734 leads to lower intracellular glycine levels, because less glycine is transported from the extracellular space. This forces the cells to compensate by synthesizing glycine from other sources, primarily serine. Serine is synthesized in glial cells, mostly astrocytes, from glucose via the phosphorylated pathway by the sequential reactions of three enzymes: 3-phosphoglycerate dehydrogenase (Phgdh), phosphoserine aminotransferase (Psat1), and phosphoserine phosphatase (Psph).^49^ Psat1 uses glutamate as an amino donor, then an increased synthesis of serine^49^ as a glycine precursor may be reflected in a decrease in the substrate pool. Thus, when GlyT1 is inhibited by SSR504734, cells may compensate by producing glycine from serine using glutamate as an amino donor in the phosphoserine pathway. This could potentially lead to a decrease in glutamate levels. Since astrocytes are involved in the uptake of glutamate and its conversion to glutamine for secretion and reuptake by neurons,^50^ substrate deficiency may limit glutamine availability for other cellular processes. Adenosine depletion after treatment with SSR504734 can be explained by limiting adenine synthesis in response to a lower availability of glycine. Glycine, aspartate, and two glutamine molecules are required for the synthesis of inosine monophosphate (IMP), the precursor of adenine. It may be that to preserve the pool of amino acids, cells decreased adenine synthesis. In subchronic exposure to the GlyT1 inhibitor, glutamate and adenosine levels were unchanged, which reflect adaptation of the metabolic system to glycine deficiency. Since de novo synthesis of serine and glycine is upregulated during any amino acid deficiency,^51,52^ such changes are expected to occur after chronic inhibition of glycine uptake. Future studies will show whether chronic inhibition of GlyT1 leads to the induction of transcription factors associated with reviving amino acid metabolism, which would explain the lack of changes in glutamate or adenosine levels. It is also noteworthy that the primary challenge in interpreting metabolite levels in complex tissue samples lies in the differential responses of the specific cell types. For instance, a decrease in glutamate levels in the brainstems of animals treated with SRR504734 does not induce any behavioral changes, as this deficiency may not be associated with neurons or the synaptic space but rather with effects on other cell types. Hence, it would be valuable to investigate how GlyT1 inhibition affects amine and neurotransmitter levels in various cell types, i.e., neurons and glial cells.

It is widely known that (neuro)inflammation is highly implicated in the pathophysiology of epilepsy. The interplay between neuroinflammation and epilepsy appears to be bidirectional as seizures may lead to inflammatory processes or, conversely, conditions associated with neuroinflammation could contribute to seizures and epileptogenesis.^53^ Glycine, as a nonessential amino acid, is a chemical mediator that supports the function of many cell types, including cells of the immune system, and thus is considered to have immunomodulatory effects.^54^ Through activation of GlyRs, glycine induces hyperpolarization of the cell membrane in the target cell, resulting in a reduced response to pro-inflammatory stimuli. By acting on a variety of effector cells, such as monocytes, macrophages, neutrophiles, or T lymphocytes,^55,56^ glycine can decrease their sensitivity to pro-inflammatory stimuli and reduce the production of pro-inflammatory cytokines such as TNF-α or IL-6 by inhibiting the NF-κB/Iκκ pathway.^57^ Moreover, macrophages express GlyT1,^58^ and thereby, inhibiting GlyT1 with SSR504734 in these cells may alter glycine uptake, influencing cell signaling pathways involved in cytokine production and release. Low concentrations of glycine in the body result in the occurrence of inflammation,^9^ while the effects of its excessive accumulation in tissues are not fully understood. SSR504734 increases glycine’s level, which translates into the activity of immunocompetent cells in the body and the production and secretion of cytokines.^59^ Analysis of our results showed elevated levels of pro-inflammatory cytokines such as TNF-α, Il-6, and Il-1β in the serum of mice treated with SSR504734. TNF-α has a particular importance in the development of inflammatory processes, as it induces the Th1 cellular response phenotype and participates in the regulation of the immune response of the body exposed to negative internal stimuli,^60^ such as excessive glycine accumulation following the use of SSR504734. It is worth mentioning that TNF-α equally stimulates the release of Il-6 and Il-1 from macrophages in the body’s response to a damaging stimulus.^61^ Thus, the observed increase in these cytokine levels may be the result of the stimulation of general immune mechanisms. IL-6, a pro-inflammatory cytokine released by circulating immune cells, fibroblasts, and endothelial cells, causes the initiation of the acute phase reaction and the synthesis of cytokines in hepatocytes.^62^ However, the results of our study did not show any changes in the cytokine levels in liver tissue. Therefore, it cannot be concluded that the increase in these cytokine levels in serum is the result of the development of a rapidly transient inflammation induced by the action of the administered compound and the release of these cytokines from other tissues and organs, e.g., the CNS, in which glycine transport is impaired. It is noteworthy that IL-6 affects the differentiation of T lymphocytes toward a Th2 phenotype and the secretion of anti-inflammatory cytokines.^63^ Comparing high concentrations of IL-6 with high concentrations of IL-10 in serum, it should be assumed that it participates in the processes of immune response modulation. SSR504734 also increased the concentration of IL-1β which is considered to have a proconvulsant effect. However, in response to seizures, IL-1β may induce the synthesis of IL-1Ra which, in turn, reduces the pro-inflammatory effects of IL-1β. Of note, IL-1Ra has been reported to have an antiseizure effect in various seizure models.^64^ Furthermore, the use of a GlyT1 inhibitor may result in the occurrence of oxidative stress and, consequently, the production of reactive oxygen species. This may be confirmed by the increase in serum TLR4 concentration, which is not only involved in promoting the Th1-type inflammatory response but also in orchestrating the inflammatory response under stress conditions.^65^ The high concentration of IL-10 found in the serum of mice receiving SSR504734 also supports the immunoregulatory processes in response to this compound. IL-10 exerts an antagonistic effect on TNF-α, modifies the immune response by directly affecting T cells, inhibits the synthesis of pro-inflammatory cytokines, and limits the Th1-type immune response.^66^ Importantly, numerous animal and human studies show that seizures are associated with significant alterations in cytokines production. SSR504734 increased the serum level of pro-inflammatory mediators, suggesting that it may have a deteriorating effect on epileptic seizure. However, such a conclusion may be an oversimplification. First, SSR504734 increased the level of pro-inflammatory markers in serum, not in the liver, which does not suggest a general peripheral inflammation. Moreover, since we did not investigate its effect on inflammatory markers in the brain, we cannot assume that SSR504734 may induce neuroinflammation and deteriorate the course of the disease. Although we studied its effect in acute seizure models, not in models of epilepsy, we did not observe negative effects on seizure susceptibility; i.e., SSR504734 did not decrease seizure thresholds in mice. It is also noteworthy that the interplay between neuroinflammation and epilepsy is much more complex. While pro-inflammatory cytokines like IL-1β, IL-6, and TNF-α are generally associated with increased seizure susceptibility, some studies suggest that they may also have neuroprotective effects.^67−70^ Interestingly, the administration of some anti-inflammatory agents (e.g., aspirin, celecoxib, nimesulide, indomethacin) was shown to exacerbate seizures, increase mortality, or induce neurotoxicity in experimental models.^71−74^ Furthermore, neuroinflammation may also play a positive role in CNS recovery.^75^ In summary, it should be emphasized that the release of pro- and anti-inflammatory cytokines following a 14-day treatment with SSR504734 indicates the activation of both cellular and humoral mechanisms of the immune response and the processes involved in maintaining the Th1/Th2 balance. Full understanding of the properties of SSR504734 requires further research conducted at the cellular level, particularly the evaluation of cytokine expression as well as its effect on metabolic processes occurring in cells. Moreover, its effects on the inflammatory status in brain structures should be investigated.

Finally, we also assessed the pharmacokinetic profile in mice and the in vitro ADME-Tox properties of SSR504734. The pharmacokinetic parameters of SSR504734 and, in particular, good brain penetration (brain-to-serum AUC_last_ ratio >3) together with a sustained CNS exposure support its potential utility in treating neurological disorders. The *C*_max_ of the compound in the brain tissue equal to 5150 ng/mL (assuming brain tissue density of ∼1 g/mL according to https://www.aqua-calc.com/calculate/weight-to-volume/substance/brain), substantially exceeding the IC_50_ for GlyT1 inhibition which is 7.8 (±2.6) ng/mL (obtained in an in vitro assay),^27^ indicates a near-maximal target engagement at the *C*_max_ in the brain. As SSR504734 concentrations both in serum and brain tissue were observed up to 8 h, a prolonged action of this compound in the CNS may be anticipated after i.p. administration to mice at a dose of 30 mg/kg. The prolonged MRT and high AUC_last_ of SSR504734 in the brain further confirms its potential for maintaining therapeutic concentrations, aligning with the observations in Figure 1. The wide tissue distribution of the compound (Figure 2) raises further considerations. High concentrations in the lungs and spleen suggest these organs as significant drug reservoirs, with possible implications for off-target effects. The short *t*_max_ (5–15 min) in peripheral tissues reflects the rapid systemic distribution. Moderate distribution to adipose tissue indicates potential drug sequestration, which could affect the long-term clearance. The calculated pharmacokinetic parameters, including the moderate CL/F and high Vz/F, implicate the need for further studies to refine dosing regimens and safety profiles for multiple administration of SSR504734. In ADME-Tox in vitro studies, compound SSR504734 showed relatively good drug-like properties. An excellent permeability and a very high metabolic stability should be underlined. The neurotoxicity studies show a moderate effect on neuroblastoma SH-SY5Y cells. However, the fraction bound to plasma proteins comparable to warfarin and the potential risk of hepatotoxicity at higher doses may be limitations in further development of this compound.

Given the limitations observed, it would be advisible to test other selective GlyT1 inhibitors in seizure and epilepsy models as well. Agents with greater potency than SSR504734 may offer improved efficacy. However, evaluating new inhibitors requires extensive in vitro and in vivo studies, including pharmacokinetic profiling. Only comprehensive results will allow for a reliable pharmacokinetic/pharmacodynamic analysis, enabling a comparison of different molecules. The lack of detailed pharmacokinetic and tissue distribution studies on other GlyT1 inhibitors in the same species makes direct comparisons regarding their pharmacokinetic properties unfeasible. Given this gap in the literature, SSR504734 presents a unique opportunity for further characterization, particularly in relation to clinically tested GlyT1 inhibitors, such as bitopertin, iclepertin, PF-03463275, and synapsinae, which have been explored for their therapeutic potential in neuropsychiatric disorders. Bitopertin (RG1678), developed by Roche, was investigated for its ability to ameliorate negative symptoms of schizophrenia but failed to demonstrate efficacy in phase III trials.^76^ Iclepertin (BI 425809), from Boehringer Ingelheim, is currently undergoing phase III trials as a cognitive enhancer in schizophrenia.^59^ PF-03463275 (Pfizer) was evaluated for its potential to enhance cognitive training and promote neuroplasticity in schizophrenia.^77^ Another drug candidate, SNG-12 (synapsinae), developed by SyneuRx, has reached phase III trials for depressive disorders and suicidal ideation, while phase II studies have examined its potential in psychotic disorders and dementia.^78^ Given that these compounds have already undergone clinical testing for other conditions, assessing their potential efficacy in epilepsy seems reasonable.

In summary, we showed that the selective GlyT1 inhibitor SSR504734 affects exclusively the MES-induced tonic hindlimb extension in mice, which suggests its potential efficacy against generalized tonic-clonic seizures. Multitarget treatment (either by multitarget antiseizure medications or by combinations of drugs with multiple targets) is a common approach in pharmacotherapy in epileptic patients due to the heterogeneous and complex nature of epilepsy.^79^ None of the currently available antiseizure drugs directly affects GlyT1 and/or glycinergic neurotransmission. Therefore, additional studies should be considered to investigate the influence of SSR504734 on the activity of various antiseizure drugs to confirm whether inhibition of GlyT1 may be complementary to other mechanisms of action of antiseizure drugs and to validate new target combinations. Next, our findings indicate that inhibition of GlyT1 by SSR504734 may affect amino acid metabolism in the brain leading to alteration in synthesis of, e.g., glutamate and adenosine. The observed changes in the inflammatory markers in serum may suggest possible influence of SSR504734 on the brain’s innate immune system, which deserves further attention. We also found that SSR504734 is well absorbed after i.p. dosing to mice and slowly eliminated from serum and tissues. Importantly, it revealed a high brain exposure that should result in a prolonged therapeutic effect after both single and multiple dosing. In addition, the ADME-Tox data demonstrated an excellent metabolic stability in human liver microsomes, a very high passive permeability through the artificial membrane in the PAMPA test, and moderate neurotoxicity. On the other hand, tests on hepatoma HepG2 cells may indicate the potential hepatotoxic effect, however, only in higher concentrations. More studies are required to better describe the effects mediated by SSR504734 and to evaluate GlyT1 as a possible novel molecular target in the treatment of epilepsy.

## Data Availability

The data that support the findings of this study are openly available in Zenodo repository (https://doi.org/10.5281/zenodo.14925596).

## Supporting Information Available

Synthesis of SSR504734, experimental conditions used in the GlyT1 binding assays; effect of a 14-day treatment with SSR504734 on glutamate, GABA, adenosine, and glycine concentrations in brain structures; effect of 14-day treatment with SSR504734 on the level of inflammatory markers in the liver; UPLC spectra after 120 min incubation of SSR504734 with HLMs; MS analysis of SSR504734; MS analysis and the most probable structure of metabolites M1–M3; effect of SSR504734 on hepatoma HepG2 and neuroblastoma SH-SY5Y cell line viability; time-course effect of SSR5504734 on neuromuscular strength and motor coordination in mice; effect of acute and 14-day treatment with SSR504734 on neuromuscular strength and motor coordination in mice; and summary of statistical analysis for Figure 5 and Figures S1–S2 (PDF)
