# Supplementary material for: Effect of SSR504734, a Selective Glycine Transporter Type 1 Inhibitor, on Seizure Thresholds, Neurotransmitter Levels, and Inflammatory Markers in Mice
Source: ACS Chem Neurosci. 2025 Feb 27;16(6):1210–26. doi: 10.1021/acschemneuro.5c00039 (PMC11926788; doi:10.1021/acschemneuro.5c00039)
Supplement: Supplementary file 1 — cn5c00039_si_001.pdf [file cn5c00039_si_001.pdf]

Supporting Information for

Influence of SSR504734, a selective glycine transporter type 1 inhibitor, on seizure thresholds, neurotransmitter levels, and inflammatory markers in mice

Nikola Gapińska[1,2], Piotr Wlaź[1], Elżbieta Wyska[3], Artur Świerczek[3], Krzysztof Kamiński[4], Marcin Jakubiec[4], Michał Abram[4], Katarzyna Ciepiela[4,5], Gniewomir Latacz[6], Tymoteusz Słowik[7], Dawid Krokowski[8], Łukasz Jarosz[9], Artur Ciszewski[9], Katarzyna Socała[1*]

[1]Department of Animal Physiology and Pharmacology, Institute of Biological Sciences, Maria Curie-Skłodowska University, Akademicka 19, 20–033 Lublin, Poland

[2]Doctoral School of Quantitative and Natural Sciences, Maria Curie-Skłodowska University, Weteranów 18, 20–038 Lublin, Poland

[3]Department of Pharmacokinetics and Physical Pharmacy, Faculty of Pharmacy, Jagiellonian University Medical College, Medyczna 9, 30–688 Cracow, Poland

[4]Department of Medicinal Chemistry, Faculty of Pharmacy, Jagiellonian University Medical College, Medyczna 9, 30–688 Cracow, Poland

[5]Selvita S.A., Bobrzyńskiego 14, 30–348 Cracow, Poland

[6]Department of Technology and Biotechnology of Drugs, Jagiellonian University Medical College, Medyczna 9, 30–688 Cracow, Poland

[7]Experimental Medicine Center, Medical University, Jaczewskiego 8, 20–090 Lublin, Poland

[8]Department of Molecular Biology, Institute of Biological Sciences, Maria Curie-Skłodowska University, Akademicka 19, 20–033 Lublin, Poland

[9]Department of Epizootiology and Clinic of Infectious Diseases, Faculty of Veterinary Medicine, University of Life Sciences in Lublin, Głęboka 30, 20–612, Lublin, Poland

[*]**Corresponding author**: Katarzyna Socała
Department of Animal Physiology and Pharmacology, Institute of Biological Sciences, Maria Curie-Skłodowska University, Akademicka 19, 20–033 Lublin, Poland
e-mail: katarzyna.socala@mail.umcs.pl

Table of contents

Synthesis of SSR504734 3

Scheme S1. Synthesis of target SSR504734 based on (Rousseau et al., 2015) 4

Figure S1. Effect of 14-day treatment with SSR504734 on (A) glutamate, (B) GABA, (C) adenosine, and (D) glycine concentrations in brain structures 9

Figure S2. Effect of 14-day treatment with SSR504734 on the level of inflammatory markers in the liver 10

Figure S3. UPLC spectra after 120 min incubation of SSR504734 with HLMs 11

Figure S4. MS analysis of SSR504734 11

Figure S5. MS analysis and the most probable structure of metabolite **M1** 12

Figure S6. MS analysis and the most probable structure of metabolite **M2** 12

Figure S7. MS analysis and the most probable structure of metabolite **M3** 12

Figure S8. The effect of cytostatic drug doxorubicin and SSR504734 on (A) hepatoma HepG2 and (B) neuroblastoma SH-SY5Y cell line viability after 72 h of incubation at 37°, 5% CO_2 13

Table S1. Experimental conditions used in the GlyT1 binding assays 14

Table S2. Time-course effect of SSR5504734 on neuromuscular strength and motor coordination in mice 15

Table S3. Effect of acute and 14-day treatment with SSR504734 on neuromuscular strength and motor coordination in mice 15

Table S4. Summary of statistical analysis for Figure 5 16

Table S5. Summary of statistical analysis for Figure S1 17

Table S6. Summary of statistical analysis for Figure S2 18

References 19

Synthesis of SSR504734

The final SSR504734 was synthesized following the procedure described in literature (Rousseau et al., 2015) and illustrated in **Scheme S1**. Initially, 2-Benzoylpyridine was subjected to catalytic hydrogenation using platinum oxide (PtO_2) in acetic acid (AcOH) at 30 bar. This step yielded a mixture of *erythro* and *threo* diastereomers (80:20) of phenyl-piperidine-2-yl-methanol. In the next step the *erythro* and *threo* diastereomers were separated by selective crystallization of the hydrochloride salt ethanol/diethyl ether, enriching the *erythro* isomer. After neutralization with sodium hydroxide, the *erythro* diastereomer was obtained with enhanced diastereomeric purity. Further resolution of the *erythro* racemate was achieved using (+)-(D)-di-p-toluoyl tartaric acid in an ethanol/water mixture to isolate the erythro (*R,S*) enantiomer. The *erythro* amino-alcohol (*R*,*S*) **3** was reacted with thionyl chloride ($SOCl_2$) in dichloromethane (DCM) in the presence of pyridine and (4-dimethylaminopyridine) DMAP. This controlled reaction at low temperature (-5°C) yielded sulfamidite **4** as a mixture of two diastereomers (due to the presence of chiral sulfoxide). In the next step sulfamidite **4** was oxidized using a combination of $NaIO_4$ and $RuCl_3$ in an acetonitrile-water mixture to form sulfamidate **5**. Following the oxidation, the product was isolated and used directly in subsequent steps without the need for extensive purification. Intermediate 2-chloro-3-trifluorobenzamide **7** was synthesized from 2-chloro-3-trifluorobenzoic acid **6** by its conversion to the corresponding acid chloride, followed by reaction with ammonia to form the amide. Finally the anion of 2-chloro-3-trifluorobenzamide **7**, generated using sodium *tert*-amylate in tetrahydrofuran (THF), was reacted with sulfamidate **5** in *N*-methyl-2-pyrrolidone (NMP). The resulting sulfamic acid intermediate was hydrolyzed with sulfuric acid to produce final SSR504734.

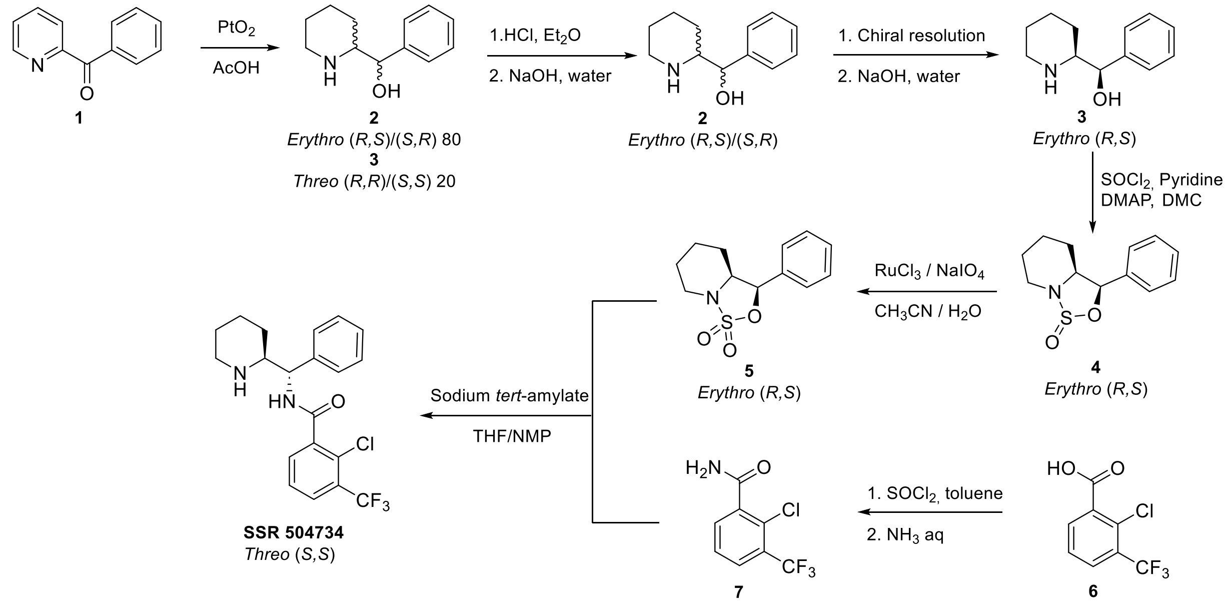

Scheme S1. Synthesis of target SSR504734 based on (Rousseau et al., 2015).

Analysis and spectral data

All chemicals and solvents were purchased from commercial suppliers and were used without further purification. Melting points (mp.) were determined in open capillaries on a Büchi 353 melting point apparatus (Büchi Labortechnik, Flawil, Switzerland) and are uncorrected. The purity and homogeneity of the compounds were assessed by thin-layer chromatography (TLC) and the gradient UPLC chromatography. TLC was performed in silica gel 60 F_{254} pre-coated aluminum sheets (Macherey-Nagel, Düren, Germany), using developing system that consisted of the following: S_1–DCM:MeOH (9:1; *v*/*v*), S_2–DCM:MeOH (9:0.5; *v*/*v*). Spots were detected by their absorption under UV light (λ = 254 nm). The UPLC and mass spectra (LC-MS) were obtained using: **Method 1:** Shimadzu (Kyoto, Japan) LCMS-2020 Single Quadrupole Liquid Chromatograph Mass Spectrometer. A Waters ACQUITY™ UPLC BEH C18 column (1.7 μm, 2.1 × 100 mm, 130 Å, column no. 186002352) was employed (Waters, Milford, CT, USA). Chromatographic conditions were as follows: column temperature 25°C, flow rate 0.5 mL/min, injection volume 3 μL, and detection wavelengths 254 nm and 230 nm. The mobile phase consisted of eluent A (water

with 0.1% v/v formic acid) and eluent B (acetonitrile with 0.1% v/v formic acid), using a gradient of 80:20 (A:B) increasing to 5:95 over a 5-minute period. **Method 2** (performed by Reach Separations, Nottingham, United Kingdom): The LCMS analysis was performed using a Waters ACQUITY™ H-Class UPLC System, C18 CSH column (1.7 µm, 2.1 × 50 mm) at 60°C (Waters, Milford, CT, USA) with the Waters Acquity UPLC PDA detector (UV detector) and QDa detector (MS detector). Conditions included a flow rate of 1.0 mL/min, an injection volume of 1.0 µL, and detection wavelengths between 210 and 400 nm. The mobile phase comprised eluent A (water with 0.1% v/v trifluoroacetic acid) and eluent B (acetonitrile), with a gradient starting at 98:2 (A:B) and increasing to 0:100 over a 2.52-minute period. **Method 3:** Waters ACQUITY™ TQD system (Waters, Milford, CT, USA) with the MS-TQ detector and UV-Vis-DAD eλ detector. The ACQUITY UPLC BEH C18, 1.7 µm (2.1 × 100 mm) column was used with the VanGuard Acquity UPLC BEH C18, 1.7 µm (2.1 × 5 mm) (Waters, Milford, CT, USA). Standard solutions (1 mg/mL) of each compound were prepared in analytical grade MeCN/water mixture (1:1; *v/v*). Conditions applied were as follows: eluent A (water/0.1% HCOOH), eluent B (MeCN/0.1% HCOOH), a flow rate of 0.3 mL/min, a gradient of 5–100% B over 10 min, and an injection volume of 10 µl. Chiral separations (performed by Reach Separations, Nottingham, United Kingdom) were carried out using a Waters UPC^2™ SFC system equipped with UV (Acquity UPC^2 PDA Detector) and QDa Detector (MS detector) (Waters, Milford, CT, USA). A Lux C4 chiral column (4.6 × 250 mm, 5 µm) was used under the following conditions: column temperature 40°C, flow rate 4 mL/min, injection volume 1.0 µL, detection wavelengths between 210 and 400 nm, and an automated back-pressure regulator (BPR) set to 125 BarG. Isocratic conditions were maintained with a mobile phase of 20:80 (EtOH:CO_2) containing 0.2% v/v NH_3. The UPLC and SFC retention times (t_R) are given in minutes. ^{1}H NMR and ^{13}C NMR spectra were obtained in a JEOL-500 spectrometer (JEOL USA, Inc. MA, USA), in $CDCl_3$ operating at 500

MHz (^{1}H NMR) 126 MHz (^{13}C NMR) or Bruker BioSpin Fourier HD 300 MHz spectrometer (Billerica, Massachusetts, USA) in $CDCl_3$ or DMSO-d_6 operating at 300 MHz. Chemical shifts are reported in δ values (ppm) relative to TMS δ = 0 (^{1}H), as internal standard. The *J* values are expressed in Hertz (Hz). Signal multiplicities are represented by the following abbreviations: d (doublet), dt (doublet of triplets), t (triple), td (triple of doublets), q (quartet), qd (quartet of doublets), m (multiplet).

The final compound SSR504734, along with all intermediate compounds, was synthesized according to the procedures described in the referenced publication (Rousseau et al., 2015). Detailed physicochemical and spectroscopic data (e.g., ^{1}H NMR, ^{13}C NMR, LC-MS, and purity assessments) for selected synthesized compounds are provided below, ensuring the characterization and confirmation of their structures.

Phenyl-piperidine-2-yl-methanol (2) *Erythro* (*R*,*S*)/(*S*,*R*) and (3) *Threo* (*R*,*R*)/(*S*,*S*)

White solid. Yield: 99.9 % (62.6 g); UPLC (Method 1) (purity > 99.9%): t_R = 1.25 min. $C_{12}H_{17}NO$ (191.27). LC-MS (ESI): *m*/*z* calcd for $C_{12}H_{17}NO$ $[M+H]^+$ 192.13, found 192.19, UPLC (Method 2) (purity > 99.9%): t_R = 0.76 min. $C_{12}H_{17}NO$ (191.27). LC-MS (ESI): *m*/*z* calcd for $C_{12}H_{17}NO$ $[M+H]^+$ 192.13, found 192.19, Chiral SFC: *erythro* (*SR*) t_R = 5.75 min (purity = 40.2%) *erythro* (*RS*) t_R = 7.22 min, (purity = 40.0%), *threo* (*RR*) t_R = 8.22 min, (purity = 10.1%), *threo* (*SS*) t_R = 12.32 min, (purity = 9.7%); ^{1}H NMR (300 MHz, $CDCl_3$) δ 1.12–1.45 (m, 3H), 1.62–1.87 (m, 3H), 1.62–2.79 (m, 1H), 2.92 (dt, *J*=11.2, 3.6 Hz, 1H), 3.25 (d, *J*=11.7 Hz, 1H), 4.55 (d, *J*=7.8 Hz, 0.2H - ***threo***), 4.91 (d, *J*=4.2 Hz, 0.6H- ***erythro***), 7.30–7.43 (m, 5H), Signals from NH (1H) and OH (1H) not visible.

Phenyl-piperidine-2-yl-methanol (2) *Erythro* (*R*,*S*)/(*S*,*R*)

White solid. Yield: 88.4 % (33.1 g); ^{1}H NMR (300 MHz, $CDCl_3$) δ 1.24–1.41 (m, 2H), 1.62–1.72 (m, 3H), 1.77–1.89 (m, 1H), 2.79 (td, *J*=11.8, 4.4 Hz, 1H), 2.94–3.03 (m, 1H), 3.29–3.42

(m, 1H), 5.09 (d, *J*=3.9 Hz, 1H), 7.30–7.42 (m, 5H), Signals from NH (1H) and OH (1H) not visible.

Phenyl-piperidine-2-ylmethanol (3) *Erythro* (*R,S*)

White solid. Yield: 32% (10.7 g); UPLC (Method 2) (purity = 98.5%): t_R = 0.76 min. $C_{12}H_{17}NO$ (191.27). LC-MS (ESI): *m/z* calcd for $C_{12}H_{17}NO$ $[M+H]^+$ 192.13, found 192.19; Chiral SFC: *erythro* (*SR*) t_R = 5.50 min (purity = 97.9%) *erythro* (*RS*) t_R = 7.35 min, (purity = 2.0%), *threo* (*RR*) t_R = 8.40 min, (purity = 0.1%), *threo* (*SS*) t_R = 12.00 min, (purity = 0.0%);^{1}H NMR (300 MHz, DMSO-d6) δ 0.95–1.09 (m, 1H), 1.12–1.28 (m, 2H), 1.33–1.49 (m, 2H), 1.55–1.65 (m, 1H), 1.67–1.77 (m, 1H), 2.34–2.46 (m, 1H), 2.84–2.94 (m, 1H), 4.32 (dd, *J*=5.6, 4.2 Hz, 1H), 5.14 (d, *J*=4.2 Hz, 1H), 7.19–7.26 (m, 1H), 7.27–7.37 (m, 4H), Signal from NH (1H) overlap with signal from DMSO. ^{1}H NMR (300 MHz, $CDCl_3$) δ 1.20–1.72 (m, 5H), 1.77–1.87 (m, 1H), 2.77 (td, *J*=12.1, 3.6 Hz, 1H), 2.96 (dt, *J*=11.3, 3.6 Hz, 1H) 3.28–3.38 (m, 1H), 5.02 (d, *J*=4.1 Hz, 1H), 7.30–7.45 (m, 5H), Signals from NH (1H) and OH(1H) not visible.

Sulfamidite (3*R*,3a*S*)-3-Phenyl-hexahydro-[1,2,3]oxathiazolo[3,4-a]pyridine 1-oxide (4)

White solid. Yield: 84% (10.4 g); TLC: R_{f1} = 0.72, R_{f2} = 0.51 (S_2) UPLC (Method 3) (purity > 98.9%): t_{R1} = 5.69, t_{R2} = 6.74 min. $C_{12}H_{15}NO_2S$ (237.31). LC-MS (ESI): *m/z* calcd for $C_{12}H_{15}NO_2S$ $[M+H]^+$ 238.08, found 238.12.

(3*R*,3a*S*)-3-Phenyl-hexahydro-[1,2,3]oxathiazolo[3,4-a]pyridine 1,1-dioxide (5)

White solid. Yield: 93% (9.9 g); TLC: R_f = 0.72 (S_1), UPLC (Method 3) (purity > 99.9%): t_R = 6.57 min. $C_{12}H_{15}NO_3S$ (253.31). LC-MS (ESI): *m/z* calcd for $C_{12}H_{15}NO_3S$ $[M+H]^+$ 254.07, found 254.17; ^{1}H NMR ($CDCl_3$) δ 0.81 (qd, *J*=11.8, 4.8 Hz, 1H), 1.07−1.83 (m, 5H), 2.64 (td, *J*=13.0, 3.2 Hz, 1H), 3.47−3.78 (m, 2H), 5.62 (d, *J*=6.3 Hz, 1H), 7.24−7.32 (m, 5H).

2-Chloro-3-trifluoromethyl-benzamide (7)

White solid. Yield: 84% (13.4 g); UPLC (Method 3) (purity > 99.5%): t_R = 4.99 min. $C_8H_5ClF_3NO$ (223.57). LC-MS (ESI): *m/z* calcd for $C_8H_5ClF_3NO$ $[M+H]^+$ 224.01, found 224.13; ^1H NMR ($CDCl_3$) δ 7.0–8.5 (m, 5H).

2-Chloro-3-methyl-*N*-((*S*)-phenyl-(*S*)-piperidin-2-yl-trifluoromethyl)-benzamide (SSR504734)

White solid. Yield: 84% (12.5 g); mp. 144.3–145.8 °C; TLC: R_f = 0.20 (S_2); UPLC (Method 3) (purity > 99.9%): t_R = 5.99 min. $C_{20}H_{20}ClF_3N_2O$ (396.83). LC-MS (ESI): *m/z* calcd for $C_{20}H_{20}ClF_3N_2O$ $[M+H]^+$ 397.12, found 397.17. ^1H NMR (500 MHz, $CDCl_3$) δ 1.31–1.36 (m, 2 H), 1.51–1.54 (m, 1 H), 1.55–1.57 (m, 1 H), 1.77–1.84 (m, 2 H), 2.45 (td, *J*=11.5, 2.7 Hz, 1 H), 2.89–2.94 (m, 2 H), 5.11 (dd, *J*=7.7, 2.9 Hz, 1 H), 7.28–7.39 (m, 7 H), 7.44 (t, *J*=7.7 Hz, 1 H), 7.71 (dd, *J*=7.6, 1.3 Hz, 1 H), 7.77 (dd, *J*=7.7, 1.4 Hz, 1 H). ^{13}C NMR (126 MHz, $CDCl_3$) δ 24.6, 25.8, 30.2, 46.9, 57.9, 60.6, 122.7 (q, *J*=273.6 Hz), 126.2, 127.1, 127.6, 128.8 (q, *J*=5.4 Hz), 128.9, 129.2, 129.6, 132.7, 139.0, 140.2, 165.9

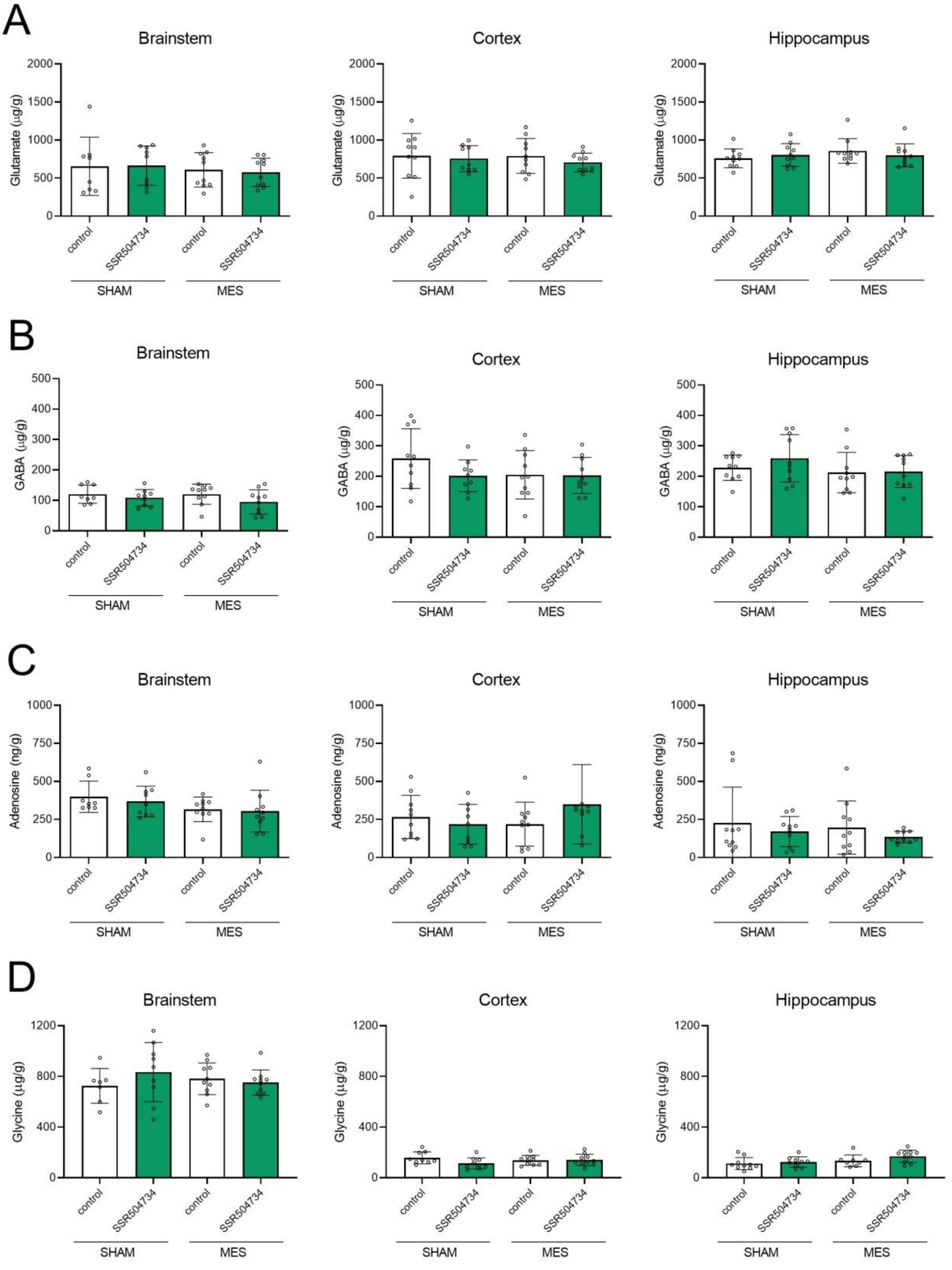

Figure S1. Effect of 14-day treatment with SSR504734 on (A) glutamate, (B) GABA, (C) adenosine, and (D) glycine concentrations in brain structures. SSR504734 and VPA were injected i.p. every 24 h for 14 days. The last administration was made 60 and 15 min before the MES stimulation, respectively. Control animals received saline. Non-stimulated (sham) animals were treated with saline or SSR504734 but they did not receive MES stimulus. Data are expressed as means ± SD. Statistical analysis: two-way ANOVA followed by Bonferroni's post hoc test.

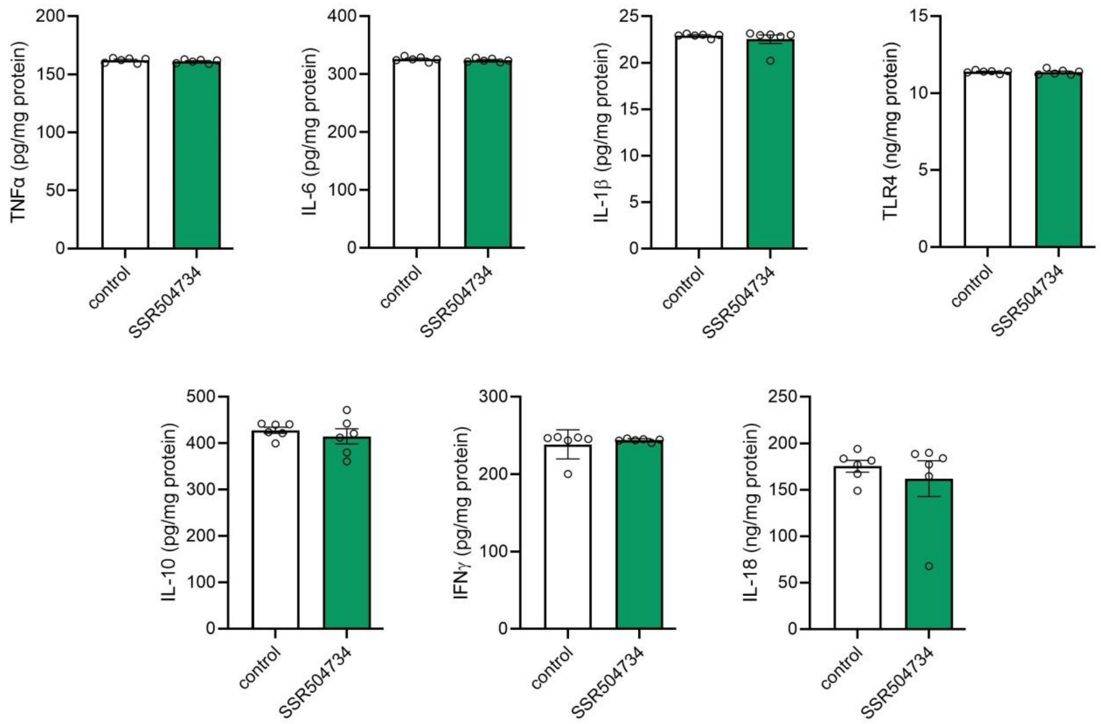

Figure S2. Effect of 14-day treatment with SSR504734 on the level of inflammatory markers in the liver. SSR504734 and VPA were injected i.p. every 24 h for 14 days. The last administration was made 60 and 15 min before the MES stimulation, respectively. Control animals received saline. Non-stimulated (sham) animals were treated with saline or SSR504734 but they did not receive MES stimulus. Data are expressed as means ± SD. Statistical analysis: unpaired Student's t-test.

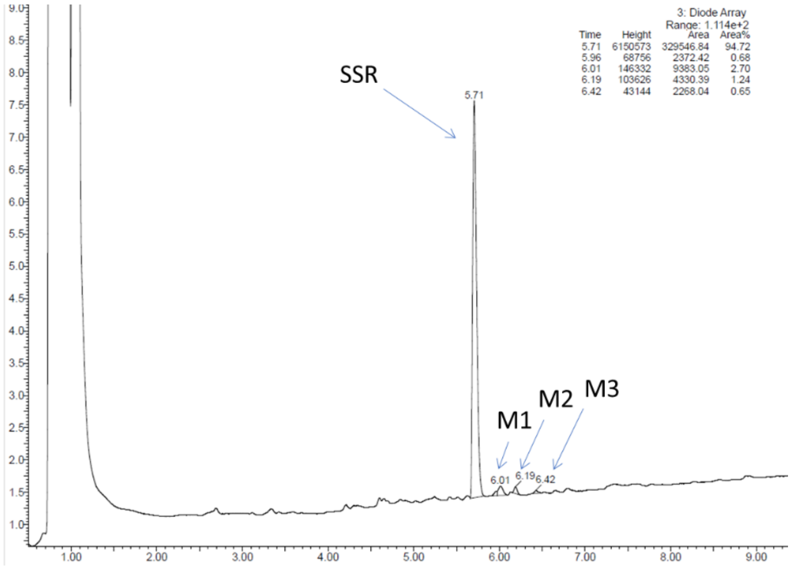

Figure S3. UPLC spectra after 120 min incubation of SSR504734 with HLMs.

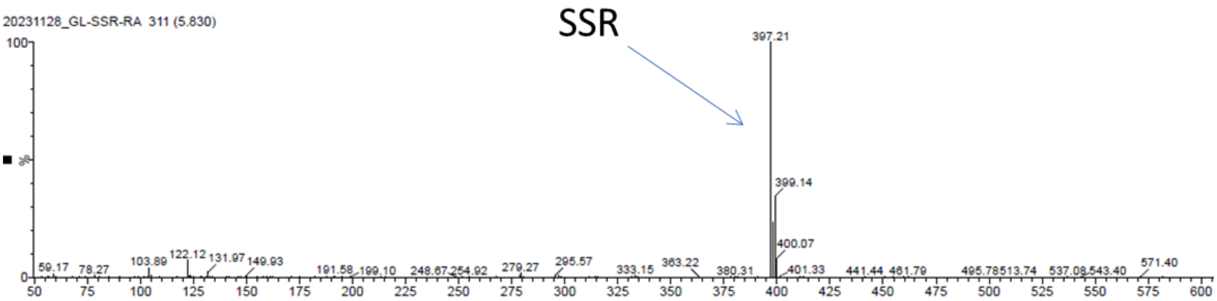

Figure S4. MS analysis of SSR504734.

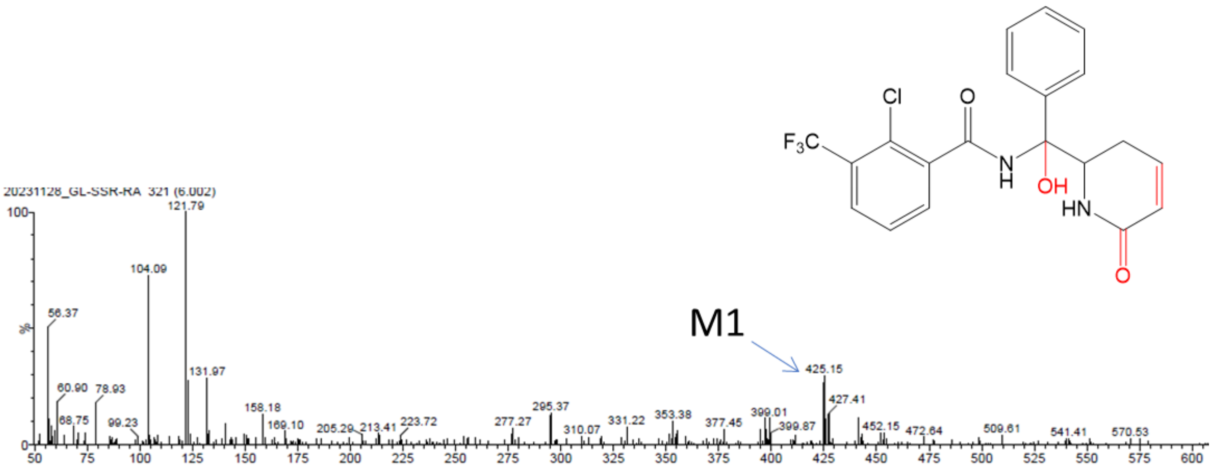

Figure S5. MS analysis and the most probable structure of metabolite **M1**.

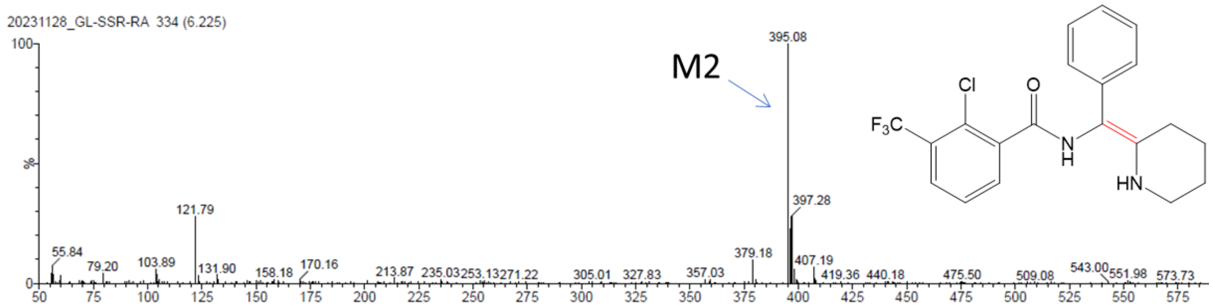

Figure S6. MS analysis and the most probable structure of metabolite **M2**.

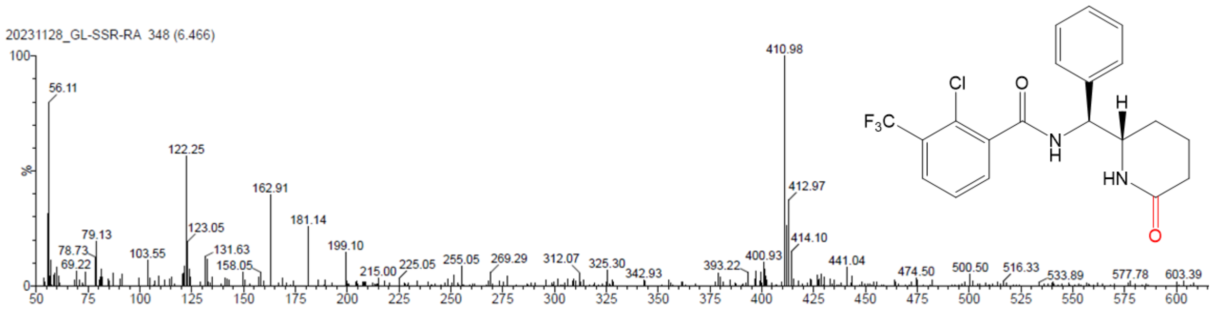

Figure S7. MS analysis and the most probable structure of metabolite **M3**.

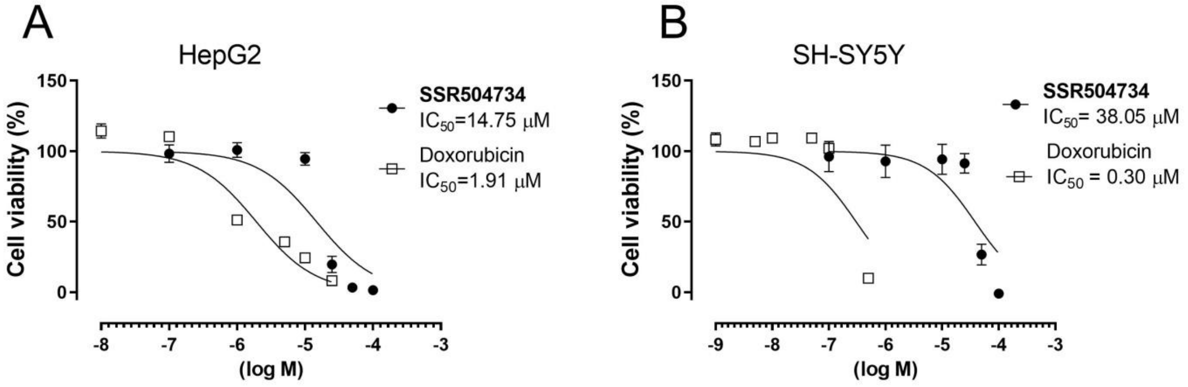

Figure S8. The effect of cytostatic drug doxorubicin and SSR504734 on (**A**) hepatoma HepG2 and (**B**) neuroblastoma SH-SY5Y cell line viability after 72 h of incubation at 37 °C, 5% CO_2. The IC_{50} values were calculated by GraphPad Prisma 8.0.

Table S1. Experimental conditions used in the GlyT1 binding assays.

Source	Ligand	Con	K_d	Non specific	Incubation	Buffer	Detection method
**Wistar rat brain cortex**	[3H] Glycine	0.05 µM	320 µM	10.0 mM glycine	15 min, 37°C	20 mM HEPES, pH 7.4, 150 mM NaCl, 5 mM KCl, 1 mM $CaCl_2$, 1 mM $MgCl_2$, 10 mM Glucose	Radioligand binding

Table S2. Time-course effect of SSR5504734 on neuromuscular strength and motor coordination in mice

Group	Neuromuscular strength (mN/g)	Impairment of motor coordination (%)
control	34.15 ± 4.97	0
SSR504734 15 min	28.92 ± 4.92*	0
SSR504734 30 min	30.33 ± 5.38	0
SSR504734 60 min	33.63± 4.94	0
SSR504734 120 min	33.49 ± 3.76	8.33
SSR504734 240 min	32.13 ± 3.42	0

SSR504734 was dissolved in normal saline and injected i.p. at the dose of 30 mg/kg. Control animals received saline. The data are expressed as the mean ± SD of grip strengths measured in millinewtons per gram of mouse body weight (mN/g) from the grip-strength test assessing skeletal muscle strength in mice and as the percentage of animals exhibiting motor coordination impairment in the chimney test. Each experimental group consisted of 12 animals. The grip-strength test results were analyzed using a one-way ANOVA with Dunnett's post hoc test, while the data from the chimney test were statistically evaluated using Fisher's exact probability test. *$p<0.05$ vs. control group.

Table S3. Effect of acute and 14-day treatment with SSR504734 on neuromuscular strength and motor coordination in mice

Group	Neuromuscular strength (mN/kg)	Impairment of motor coordination (%)
**acute treatment**		
control	28.85 ± 5.95	0
VPA (150 mg/kg)	29.73 ± 4.60	8.33
SSR 504734 (10 mg/kg)	31.15 ± 5.55	0
SSR 504734 (30 mg/kg)	31.00 ± 9.46	0
SSR 504734 (50 mg/kg)	30.31 ± 5.08	33.3
**14-day treatment**		
control	24.30 ± 4.36	0
VPA (150 mg/kg)	22.27 ± 4.84	0
SSR 504734 (3 mg/kg)	25.02 ± 5.35	0
SSR 504734 (10 mg/kg)	23.48 ± 5.98	0
SSR 504734 (30 mg/kg)	27.76 ± 6.00	0

In acute studies, SSR504734 and sodium valproate (VPA; positive control) were injected i.p. 60 and 15 min before the test, respectively. In subchronic studies, SSR504734 and VPA were injected i.p. every 24 h for 14 days. The last administration was made 60 and 15 min before the tests, respectively. Control animals received saline. Each experimental group consisted of 12 animals. The grip-strength test results were analyzed using a one-way ANOVA with Dunnett's post hoc test, while the data from the chimney test were statistically evaluated using Fisher's exact probability test.

Table S4. Summary of statistical analysis for Figure 5.

Neurotransmitter	Brain structure	Interaction	SSR504734	Seizures
Glutamate	Brainstem	F(1,27)=3.22 p= 0.08	F(1,27)=9.33 p=0.01	F(1,27)=4.31 p=0.05
	Hippocampus	F(1,25)=2.35 p=0.14	F(1,25)=0.46 p=0.50	F(1,25)=1.55 p=0.22
	Cortex	F(1,27)=0.07 p=0.79	F(1,27)=3.66 p=0.07	F(1,27)=0.02 p=0.89
GABA	Brainstem	F(1,27)=0.27 p=0.61	F(1,27)=4.59 p=0.04	F(1,27)=1.56 p=0.22
	Hippocampus	F(1,26)=2.43e-003 p=0.96	F(1,26)=5.56 p=0.03	F(1,26)=2.43e-003 p=0.96
	Cortex	F(1,27)=2.27 p=0.14	F(1,27)=0.02 p=0.89	F(1,27)=0.20 p=0.66
Adenosine	Brainstem	F(1,27)=4.38 p=0.05	F(1,27)=8.10 p=0.01	F(1,27)=1.72 p=0.20
	Hippocampus	F(1,24)=1.50 p=0.23	F(1,24)=0.10 p=0.75	F(1,24)=2.38 p=0.14
	Cortex	F(1,27)=2.84 p=0.10	F(1,27)=4.67 p=0.04	F(1,27)=3.32 p=0.08
Glycine	Brainstem	F(1,27)=1.16 p=0.29	F(1,27)=2.93 p=0.10	F(1,27)=1.55 p=0.22
	Hippocampus	F(1,25)=3.04 p=0.09	F(1,25)=0.83 p=0.37	F(1,25)=2.05e-003 p=0.97
	Cortex	F(1,27)=4.17e-003 p=0.95	F(1,27)=1.03 p=0.32	F(1,27)=12.63 p=0.001

Table S5. Summary of statistical analysis for Figure S1.

Neurotransmitter	Brain structure	Interaction	SSR504734	Seizures
Glutamate	Brainstem	F(1,33)=0.06 p= 0.81	F(1,33)=0.02 p=0.90	F(1,33)=0.59 p=0.45
	Hippocampus	F(1,36)=1.18 p=0.28	F(1,36)=0.02 p=0.89	F(1,36)=0.98 p=0.33
	Cortex	F(1,35)=0.12 p=0.73	F(1,35)=0.80 p=0.38	F(1,35)=0.14 p=0.72
GABA	Brainstem	F(1,33)=0.40 p=0.53	F(1,33)=2.98 p=0.09	F(1,33)=0.39 p=0.54
	Hippocampus	F(1,36)=0.51 p=0.48	F(1,36)=0.79 p=0.38	F(1,36)=2.31 p=0.14
	Cortex	F(1,35)=1.29 p=0.26	F(1,35)=1.47 p=0.23	F(1,35)=1.17 p=0.29
Adenosine	Brainstem	F(1,33)=0.08 p=0.78	F(1,33)=0.36 p=0.55	F(1,33)=4.15 p=0.05
	Hippocampus	F(1,36)=2.52e-003 p=0.96	F(1,36)=1.43 p=0.24	F(1,36)=0.45 p=0.51
	Cortex	F(1,35)=2.43 p=0.13	F(1,35)=0.52 p=0.48	F(1,35)=0.55 p=0.46
Glycine	Brainstem	F(1,32)=1.75 p=0.2	F(1,32)=0.57 p=0.46	F(1,32)=0.06 p=0.81
	Hippocampus	F(1,33)=0.56 p=0.46	F(1,33)=2.47 p=0.13	F(1,33)=4.89 p=0.03
	Cortex	F(1,34)=2.81 p=0.10	F(1,34)=1.99 p=0.17	F(1,34)=0.08 p=0.78

Table S6. Summary of statistical analysis for Figure S2.

Figure S2	Student's t-test
TNFα	t=0.90, df=10, p=0.39
IL-6	t=0.99, df=10, p=0.35
IL-1β	t=0.82, df=10, p=0.43
TLR4	t=0.30, df=10, p=0.77
IL-10	t=0.75, df=10, p=0.47
IFN-γ	t=0.70, df=10, p=0.50
IL-18	t=0.66, df=10, p=0.52

References

Rousseau J-F, Chekroun I, Ferey V and Labrosse JR (2015) Concise Preparation of a Stable Cyclic Sulfamidate Intermediate in the Synthesis of a Enantiopure Chiral Active Diamine Derivative. *Organic Process Research & Development* **19**:506-513.
